# The excess volatility puzzle explained by financial noise amplification from endogenous feedbacks

**DOI:** 10.1038/s41598-022-20879-0

**Published:** 2022-11-07

**Authors:** Alexander Wehrli, Didier Sornette

**Affiliations:** 1 Department of Management, Technology, and Economics, ETH Zurich, Zurich, 8092 Switzerland; 2 Swiss National Bank, Boersenstrasse 15, 8001 Zurich, Switzerland; 3 Institute of Risk Analysis, Prediction and Management (Risks-X), Southern University of Science and Technology, Shenzhen, 518055 China; 4 Swiss Finance Institute, c/o University of Geneva, 40 blvd. Du Pont d’Arve, 1211 Geneva 4, Switzerland

**Keywords:** Statistics, Statistical physics, Applied mathematics

## Abstract

The arguably most important paradox of financial economics—the *excess volatility puzzle*—first identified by Robert Shiller in 1981 states that asset prices fluctuate much more than information about their fundamental value. We show that this phenomenon is associated with an intrinsic propensity for financial markets to evolve towards instabilities. These properties, exemplified for two major financial markets, the foreign exchange and equity futures markets, can be expected to be generic in other complex systems where excess fluctuations result from the interplay between exogenous driving and endogenous feedback. Using an exact mapping of the key property (volatility/variance) of the price diffusion process onto that of a point process (arrival intensity of price changes), together with a self-excited epidemic model, we introduce a novel decomposition of the volatility of price fluctuations into an exogenous (i.e. efficient) component and an endogenous (i.e. inefficient) excess component. The endogenous excess volatility is found to be substantial, largely stable at longer time scales and thus provides a plausible explanation for the excess volatility puzzle. Our theory rationalises the remarkable fact that small stochastic exogenous fluctuations at the micro-scale of milliseconds to seconds are renormalised into long-term excess volatility with an amplification factor of around 5 for equity futures and 2 for exchange rates, in line with models including economic fundamentals explicitly.

## Introduction

The 20$\text {th}$ century saw revolutionary developments in the field of finance. To name only a few, the advent of theories like the efficient market hypothesis^1,2^ (EMH), the capital asset pricing model and the Black-Scholes-Merton option pricing framework provided new scientific foundations to the field. The underpinning of this new world was built on the assumption that financial markets are all-powerful computational engines, able to aggregate and process the beliefs and demands of agents, equipping prices with the full set of properly processed information currently available. And indeed, the general absence of exploitable changes in the serial structure of price fluctuations seems to support this notion^3^.

However, price dynamics in modern financial markets are the result of the activities of a multitude of interconnected agents, each trading based on their imperfect information, with heterogeneous motivations and confined by specific institutional and regulatory constraints. The resulting marketplace is in its very essence a *complex system*^4–6^ and over the years, many phenomena have been documented empirically, which are at odds with the predictions of (neo-)classical theories, like the EMH. For example, the magnitudes of price fluctuations are found to exhibit intermittency and long-range correlations, in direct support of the view that markets are a complex dynamical system with persistent memories^7,8^. Furthermore, price returns exhibit large fluctuations even in the absence of external impulses^9^. This contradicts expectations for efficient market equilibria, where price fluctuations are hypothesised to always reflect external news^10^. In fact, price change distributions are found to have fat tails^11–13^ where large deviations occur much more frequently than what one would expect if the tails were generated by exogenous shocks alone. All these phenomena contribute to levels of price fluctuations that are unjustified by the pertinent news flows – a phenomenon termed the “excess volatility puzzle”^14–18^.

Why does the aggregation process that financial markets are supposed to perform give rise to these inefficiencies? One potential explanation is that the system underlying observed prices is considerably self-referential, preventing it as a whole from converging efficiently to an unbiased estimate of the fundamental value. Under this hypothesis, the macroscopic phenomenon of excessive price fluctuations is a consequence of the *reflexive*^19^ characteristics of interactions of individual agents at different scales. The realization that reflexivity, or *feedback loops*, are crucial determinants of system dynamics in economics and finance, is indeed a view that also Shiller put at the heart of his considerations around the determinants of asset price fluctuations, as emphasized in his 2013 Nobel Prize lecture^20^. Arguably, the 2008 financial crisis and the failure of orthodox economic theory to predict these events have spurred interest in understanding the complexity, feedback loops and reflexive amplification effects inherent to the economy and financial markets^21^.

Here, we propose to capture these feedback effects with a model of epidemic spreading in collective activity dynamics, where exogenous impulses diffuse and get amplified through endogenous feedback. The statistical separation of external processes from internal amplification mechanisms allows us to dissect the observed volatility of prices into an efficient component, arising from purely exogenous fluctuations, and an inefficient component, arising from endogenous feedback processes acting as noise amplification mechanisms. In this article, we apply this method to high-frequency observations of exchange rates and equity futures prices. We find that, at longer time scales, the inferred endogenous excess volatility component is substantial and largely stable. The magnitudes of excess volatility implied by our theory—an amplification factor of $\approx 2$ for foreign exchange and $\approx 5$ for equity futures—are in remarkable agreement with previous measurements in the literature that involve fundamental values (dividends, earnings, unemployment data, GDP, etc.) explicitly. We thus add to the literature by identifying a microscopic generating mechanism for the observed excess volatility. This novel perspective also illuminates that, at intraday time scales, the endogenous excess volatility can vary strongly and markets can temporarily become highly inefficient in dissipating external impulses. Like financial markets, many (complex) systems are characterized by the composition of a large number of interacting constituents under the influence of time-varying external forcing. As such, we can also expect similar microscopic feedback mechanisms at the root of excessively volatile macroscopic behavior in other domains, e.g. causing elevated effective temperatures in physical systems. Examples here include the amplification of thermal noise into effective renormalized temperatures through quenched heterogeneities in materials^22^, in organized flows in liquids^23^ and in granular media near jamming^24^.

## Relation between volatility and conditional intensities

We consider the simple setup where the logarithm of the current price $Y(t) = \log S(t)$ is given by

1 $$\begin{aligned} Y(t) = X(t) + U(t). \end{aligned}$$

The observation error *U*(*t*) contaminates the measurement of the latent log-price *X*(*t*), which represents the market’s consensus on the current fundamental value of the asset. *X*(*t*) is assumed to follow the dynamics $\mathrm {d}X(t) = a(t)\mathrm {d}t + \sigma _X(t) \mathrm {d}B(t)$, for a standard Brownian motion *B*, some locally bounded and predictable drift *a*, and an adapted càdlàg volatility process $\sigma _X$. The noise process *U*(*t*) captures features inherent to how interactions on electronic financial markets are designed, and how *S*(*t*) is constructed. It is for example customary that prices can only be expressed as multiples of some minimum price increment (the “tick size”), which gives rise to measurement error due to price discreteness. Furthermore, since electronic markets at any given moment in time are comprised of a collection of trading interests of many agents (represented by the so-called “limit order book”^25^), a reduced form model like (1) will have to define how to combine a multitude of available prices into *S*(*t*). It is for example customary to consider the *mid-price* of an asset, which is defined as the average of the lowest available price some market participant is willing to sell the asset for (the best offer/ask price), and the highest available price some participant is willing to buy (the best bid price). In this case, e.g. nonsynchronicity in revisions of the best bid and offer price may induce serial dependence in the innovations of *Y*, which are captured by *U*.

An insightful representation of the instantaneous variance of the observed price follows from the definition

2 $$\begin{aligned} \sigma ^2_Y(t) = \lim _{\Delta \downarrow 0}\Delta ^{-1}{\mathbb {E}}[(Y(t+\Delta )-Y(t))^2 \mid {\mathscr {F}}_t], \end{aligned}$$

where ${\mathscr {F}}$ is the filtration of the process. Since the squared returns are realizations of a non-negative random variable, we can express (2) as

3 $$\begin{aligned} \sigma ^2_Y(t) = \lim _{\Delta \downarrow 0}\Delta ^{-1} \int _0^\infty {\mathbb {P}}[(Y(t+\Delta )-Y(t))^2 \ge \varepsilon \mid {\mathscr {F}}_t] \mathrm {d} \varepsilon = \int _0^\infty \lim _{\Delta \downarrow 0}\Delta ^{-1} {\mathbb {P}}[(Y(t+\Delta )-Y(t))^2 \ge \varepsilon \mid {\mathscr {F}}_t] \mathrm {d} \varepsilon . \end{aligned}$$

We note that the terms being integrated in (3) can equivalently be stated as conditional intensities of counting processes $N^ \varepsilon (t)$ which count the times where the squared return exceeds a threshold of $\varepsilon$. Thus we can develop the variance process as

4 $$\begin{aligned} \begin{aligned} \sigma ^2_Y(t)&= \int _0^\infty \lim _{\Delta \downarrow 0}\Delta ^{-1} {\mathbb {P}}[N^ \varepsilon (t+\Delta ) - N^ \varepsilon (t) > 0 \mid {\mathscr {F}}_t] \mathrm {d} \varepsilon = \int _0^\infty \lambda ^ \varepsilon (t) \mathrm {d} \varepsilon , \end{aligned} \end{aligned}$$

where the $\lambda ^ \varepsilon (t)$ are the conditional intensity functions of the respective processes $N^ \varepsilon (t)$. The point of view taken here—looking at the variance as the continuous cumulation of threshold-exceedence intensities—is to the best of our knowledge novel to the literature. It however resonates with a rich strain of literature documenting multifractal properties of financial volatility^26,27^.

From (2) and the definition of the observed price, we see that

5 $$\begin{aligned} \sigma ^2_Y(t) = \sigma _X^2(t) + c(t), \end{aligned}$$

where $c(t) = 2{\mathbb {E}}[\mathrm {d}X(t)\mathrm {d}U(t) \mid {\mathscr {F}}_t] + {\mathbb {E}}[(\mathrm {d}U(t))^2 \mid {\mathscr {F}}_t]$. Thus the variance of the unobservable price *X* is recovered from observed conditional intensities as

6 $$\begin{aligned} \sigma ^2_X(t) = \int _0^\infty \lambda ^ \varepsilon (t) \mathrm {d} \varepsilon - c(t). \end{aligned}$$

In practice, rather than $\sigma ^2_X(t)$, one is then typically interested in the integrated variance $v(t) = \int _{0}^{t}\sigma ^2_X(s)\mathrm {d}s$, as this quantity is more closely related to observables^28^.

Note that expression (6) maps the key property (volatility or variance) of a diffusion process onto that of point processes^29–31^ (the conditional intensities $\lambda ^ \varepsilon (t)$). This new angle of investigation is at the origin of our fundamental explanation of the excess volatility puzzle. As shown in Supplementary Information online, section 4, the presence of *c*(*t*) only affects the magnitudes of $\sigma ^2_X(t)$ when measured through the $\lambda ^\varepsilon$, and bias correction techniques are available. Importantly, *c*(*t*) does not impact a decomposition of the $\lambda ^ \varepsilon$, which is crucial to determine endogenous factors in the intensities $\lambda ^ \varepsilon$ as we do subsequently.

## Noise amplification from endogenous feedbacks

Being the primary measure of risk in modern finance, volatility dynamics are essential for applications ranging from asset pricing, portfolio construction, hedging and pricing of derivatives, as well as determining a firm’s exposure to a plethora of risk factors. The most salient and well-documented feature of volatility is its intermittency, or *clustering*, e.g. measured by its slowly decaying temporal dependence^32^. According to (6), clustering in the volatility process must manifest itself somehow from the intensity processes of the arrivals of price fluctuations. The fundamental question that arises is whether this activity clustering is a consequence of externally caused perturbations (“exogenous”/exo), or whether it is intrinsic to financial markets and driven by its network dynamics (“endogenous”/endo). It is a well-established finding in the high frequency finance literature that price fluctuations and trading activity are considerably *self-excited*, i.e. exhibit endogenous clustering^33–36^. Not only do general price fluctuations appear to exhibit endogenous feedback, but also the arrival intensity of extreme price fluctuations^37,38^, as characterized by $\lambda ^ \varepsilon$ for $\varepsilon$ very large. As such, a key characteristic of interactions on financial markets is that they amplify any noise induced by external processes through internal mechanisms that follow as reactions to observed price changes.

Why would one expect that financial markets are replete with such self-referential dynamics in the first place? Looking at today’s highly computerized markets, its participants and practices, it quickly becomes obvious that we can expect positive feedback mechanisms at a wide range of scales. At short time scales, self-referential mechanisms are mainly due to technical aspects and automated trading practices, which create feedback loops by basing their decisions on the past evolution of the market. On intermediate time scales, e.g. herding in algorithmic trading strategies, the optimized execution of portfolio transactions and margin/leveraged trading create feedback loops. At the low frequency end of the spectrum finally, behavioral mechanisms like the human tendency to imitate come into play, creating feedback at time scales of potentially months to years. Here, we focus on endogenous processes acting at time scales up to one day.

According to classical theories, like the EMH, prices should not be influenced by these endogenous processes. Markets are hypothesized to be sufficiently fast and effective so that equilibrium prices are reached quick enough after an exogenous shock, so that endogenous processes—if they exist—disappear from the observations. In particular also, extreme events like financial crashes are, according to these theories, the signature of exogenous negative news of large impact. However, there is significant evidence that extreme events on all time scales can be the result of endogenous feedback. From “ultrafast extreme events”^39^ at sub-millisecond time scales to the formation of bubbles and financial/economic crashes over years and decades^6,40^, self-referential feedback has been linked to extreme events. As such, the quantification of such processes in financial markets—just like in other complex natural and social science systems—becomes key for the prediction and assessment of extreme events^41^.

### Epidemic model for the mid-price activity

While there is ample motivation, both theoretically and empirically, to assume that endogenous feedbacks are a relevant generating mechanism of observed activity clustering, the question considering (4) is whether this is true at all scales $\varepsilon$. In order to test whether such clustering is better described by endogenous responses or exogenous shocks, we suppose that the interactions of agents making up $\lambda ^ \varepsilon$ can be described using an epidemic branching process. This branching process encodes the cascade of reactions on the network of market participants as an external perturbation diffuses through the network. Epidemic models have been shown to be able to robustly classify collective dynamics into endogenous and exogenous signatures^42^ and in particular also gained substantial recent popularity in finance^43^.

Working on a probability space $(\Omega , {\mathscr {F}}, {\mathbb {P}})$, a general epidemic branching process^44^ of price fluctuations above some threshold can be represented with a point process $N^\varepsilon : \Omega \times {\mathscr {B}}({{\mathbb {R}}}^+) \rightarrow {{\mathbb {N}}}_0 \cup \{\infty \}$, defined as a random measure on ${{\mathbb {R}}}^+ = \{x \in {{\mathbb {R}}}| x \ge 0\}$. $N^\varepsilon$ counts the random number of points (i.e. price fluctuations above $\varepsilon$) in some Borel set $A \subseteq {{\mathbb {R}}}^+$, denoted $N^\varepsilon (A)$. For ease of notation, we write $N^\varepsilon (t) := N^\varepsilon ((0,t])$ for $t\in [0, \infty )$. We will assume that the process is orderly in the sense that $N^\varepsilon (\{t\}) \in \{0,1\}$ for all $t\in {{\mathbb {R}}}^+$, in which case $N^\varepsilon$ can be described by an ordered random sequence $(t_j)_{j\in {{\mathbb {Z}}}}$ representing all the times $s\in [0,t]$ where $\mathrm {d}N^\varepsilon (s) = N^\varepsilon (s) -\lim _{u\rightarrow s^-} N^\varepsilon (u) = 1$. The history of the process $N^\varepsilon$ is given by $\sigma (N^\varepsilon ) = ({\mathscr {F}}_t)_{t\in (0,\infty )}$ where ${\mathscr {F}}_t \subseteq {\mathscr {F}}$ is the $\sigma$-algebra generated by $N^\varepsilon$ up to and including *t*, i.e. ${\mathscr {F}}_t = \sigma (N^\varepsilon (s), s\le t)$. We will assume that the epidemic process is driven by external immigration in the form of an inhomogeneous Poisson process $N^{\varepsilon ,\mu } : \Omega \times {\mathscr {B}}({{\mathbb {R}}}^+) \rightarrow {{\mathbb {N}}}_0$ with rate $\mu ^\varepsilon : [0, \infty ) \rightarrow [0, \infty )$. Furthermore, let $N^{\varepsilon ,\theta } : \Omega \times {\mathscr {B}}({{\mathbb {R}}}^+) \rightarrow {{\mathbb {N}}}_0$ be a Poisson cluster process where the cluster generated by the some point of $N^{ \varepsilon ,\mu }$ at $s<t$ is distributed according to an inhomogeneous Poisson process with intensity $\gamma (s)g(t-s)$. The memory kernel $g:[0,\infty )\rightarrow [0,\infty )$ satisfies $\int _{0}^\infty g(t)\mathrm {d}t = 1$. The quantities $\gamma (s)\ge 0$ are the “fertilities” of exogenous price fluctuations, having a density $\gamma (s) \sim d$ with mean ${\bar{\gamma }} = \int _{0}^{\infty }d(\gamma )\gamma \mathrm {d}\gamma < \infty$. Moreover, in order to encode endogenous responses of the system, let $h:[0,\infty )\rightarrow [0,\infty )$, $\int _{0}^\infty h(t)\mathrm {d}t = 1$ and $\eta (s)\ge 0$, $\eta (s) \sim f$ with ${\bar{\eta }} = \int _{0}^{\infty }f(\eta )\eta \mathrm {d}\eta < \infty$. The full epidemic model is then given by the Galton-Watson cluster process $N^{ \varepsilon }$ with Poisson offspring processes having intensities $\eta (s)h(t-s)$, based on immigrants $N^{ \varepsilon ,\theta }$ at times $s<t$.

For the full filtration ${\mathscr {F}} = \sigma (N^{\varepsilon ,\mu }, N^{\varepsilon })$, the described point process has ${\mathscr {F}}$-conditional intensity function

7 $$\begin{aligned} \lambda ^ \varepsilon (t) = \mu ^\varepsilon (t) + \int _0^t\gamma (s) g(t-s)\mathrm {d}N^{ \varepsilon ,\mu }(s) + \int _0^t\eta (s) h(t-s)\mathrm {d}N^{ \varepsilon }(s). \end{aligned}$$

With respect to the filtration ${\mathscr {F}} = ({\mathscr {F}}_t)_{t\in (0, \infty )}$, the conditional intensity function can be interpreted as a conditional hazard function, i.e. $\lambda ^ \varepsilon (t) = \lim _{\Delta \downarrow 0}\Delta ^{-1}{\mathbb {E}}[N^{ \varepsilon }(t+\Delta ) - N^{ \varepsilon }(t) \mid {\mathscr {F}}_{t-}]$, as required by (4). The epidemic model (7) allows for three kinds of clustering:

(A) Deterministic exogenous clustering through $\mu ^\varepsilon$, capturing activity clusters that are *not* due to epidemic effects.
(B) Stochastic exogenous clustering through *g*, describing activity bursts that follow as direct responses to some exogenously induced fluctuation (a “shot noise” response).
(C) Stochastic endogenous clustering through *h*, encoding the distribution of waiting times between some price fluctuation and subsequent endogenous reactions (in the form of other price fluctuations) to this event in the epidemic cascade.

The branching representation of this process is that $\mu ^ \varepsilon$ induces immigrants according to an inhomogeneous Poisson process. All immigrants trigger a single generation of offspring (“reactions”) with Poisson intensity $\gamma (s)g(t-s)$. In addition, all existing points trigger a first generation of endogenous offspring with intensity $\eta (s)h(t-s)$, which in turn trigger their own generation of offspring in the same manner, and so on. The collection of all these independent Poisson processes forms the process $N^\varepsilon$. The branching coefficients ${\bar{\gamma }}$ and ${\bar{\eta }}$ define the expected number of future price fluctuations that are triggered by some exogenous (${\bar{\gamma }}$) or arbitrary (${\bar{\eta }}$) price fluctuation. ${\bar{\eta }}$ is usually referred to as the *branching ratio* of the epidemic process, and assumes the role of a control parameter of the system. For $0 \le {\bar{\eta }} < 1$, the process is *subcritical* and the activity generated by some initial immigrant event dies out with probability one. At ${\bar{\eta }} = 1$, the process is *critical*, i.e. constantly operating at the brink of explosively diverging dynamics^45^. For ${\bar{\eta }} > 1$, the process has a finite probability to explode to an infinite number of events^46^.

### The “endo-exo” problem—a universal challenge in science

**Figure 1:**
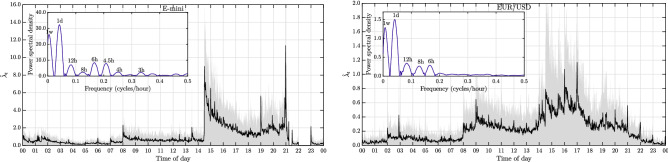
Shows intraday unconditional intensity estimates ${\hat{\lambda _t}}$ of price fluctuations (in sec$^{-1}$) from^36^, for the E-mini S&P 500 futures contract (left) and the EUR/USD exchange rate (right), obtained from a histogram estimator computed over one minute bins and averaged across days in the sample. The grey shaded areas indicate [10%, 90%] percentiles across the days, highlighting the significant day-to-day variations in the activity profile. In the insets, a smoothed power spectral density estimate for the unconditional intensity is provided, indicating the presence of periodicities in the price fluctuations at various frequencies.

In order to quantify the importance of feedback effects in general system dynamics such as (7), one faces the difficult task of decomposing aggregated dynamics into endogenous and exogenous processes—a task that has been called the “endo-exo problem”^35,47^. Since endogenous feedbacks are relevant in many scientific disciplines, it is unsurprising that their statistical identification lies at the heart of the respective domains. For example, consider the problem in seismology of determining if an earthquake is a mainshock or an aftershock, or in physics when determining if particles are interacting, or in a heterogeneous field. In epidemiology, the basic incidence of diseases is followed by contagious outbreaks. In social systems, there is e.g. the question if a surge in the popularity of some content is due to it “going viral”, or external driving. In neurophysiological studies, one finds an intricate interplay of endogenous neuronal excitation/inhibition with circadian rhythms. Many other examples exist, and a common theme in their statistical analysis is that the solution of the decomposition is often plagued by spurious inference issues, because external perturbations exhibit significant heterogeneity that is easily misidentified as endogenous memory^35,36^. In financial markets, these exogenous heterogeneities are *major* features of observed dynamics. At intraday horizons, where we intend to apply our study, there are e.g. seasonalities arising from the overlap of global trading hours, or activity bursts from scheduled economic data releases and unscheduled news, which together with endogenous processes result in complex overall activity dynamics. Moreover, these activity profiles exhibit significant day-to-day variation, as illustrated in Fig. 1. As such, a robust inference scheme is essential. Here, our solution to extract endogenous processes from these complex dynamics is based on an Expectation Maximization (EM) algorithm^48^, which adaptively learns the branching structure of the epidemic model we use to represent the collective activity process resulting from the interactions of all agents in a market. The key ingredient in the EM scheme is to use a flexible and adaptive estimator for the exogenous noise process $\mu ^\varepsilon$, so that non-stationarities are detected and controlled for properly. See Supplementary Information online, section 1 for details on the inference scheme. In order to calibrate models where ${\bar{\gamma }} > 0$, the estimation procedure needs to be extended to a Monte Carlo EM (MCEM) algorithm^44^, which is outlined in the Supplementary Information online, section 1.4.

### Determining the dominant clustering mechanism

The key question that remains in light of (7) is which of the clustering mechanisms are relevant empirically. Due to the structure of the model, this can be determined by testing nested models. Here we choose to use information criteria for this purpose. A description of the test procedure is given in Supplementary Information online, section 1.4 and detailed results from a study performed on randomly selected days from the sample are reported in Supplementary Information online, section 2. According to this procedure, the predominant model selected across thresholds $\varepsilon$ is a process with deterministic exogenous clustering (A) and stochastic endogenous clustering (C). Our analysis thus suggests that the complexity provided by an appropriately flexible and adaptive deterministic immigration intensity is sufficient, compared to adding local stochastic exogenous clustering (B). The main conclusion is that the dominant activity in financial markets is in fact *endogenous*. We also find that endogenous clustering with heavy-tailed memory frequently prevails over endogenous clustering with short-tailed (exponential) memory, in line with the persistence of volatility documented empirically^49^. Moreover, the arrival processes of large returns appear to be associated with higher—at times even (super)critical—estimates of ${\bar{\eta }}$. This is in line with models from financial risk management^37,38^, where ${\bar{\eta }} > 1$ is a frequent finding. Based on these results, we conclude that an epidemic model with $\gamma (s) \equiv 0$ and heavy-tailed endogenous memory

8 $$\begin{aligned} h(t) \propto (\tau _0 + t)^{-(1+\alpha )} \end{aligned}$$

is most appropriate to describe the microscopic activity of the financial markets considered here. To speed up estimation, we will model (8) using an approximate Pareto distribution^34^. It is clear that the tests conducted here are far from a systematic study of self-excitation in extreme returns, which is out of the scope of this paper, but merits future analysis. Such a study could potentially also help reconcile the apparent paradox that volatility profiles around large volatility fluctuations are in line with predictions of a critical system with memory^50^, whereas modelling overall market activity using self-excited processes strongly rejects a criticality hypothesis^35,36^.

## Microscopic activity and the “excess volatility puzzle”

To gain further traction on the integral $\int _{0}^{\infty }\lambda ^\varepsilon (t)\mathrm {d}\varepsilon$ in (6) for empirical purposes, we will assume that $\lambda ^\varepsilon$ can be decomposed as a sum of intensities for different amplitudes *m* of the squared returns as

9 $$\begin{aligned} \lambda ^ \varepsilon (t) = \int _{ \varepsilon }^{\infty }\lambda (t,m)\mathrm {d}m. \end{aligned}$$

In the following, it becomes clear that the realisations of *m* at each point in time play the role of “marks” of the point process. We assert accordingly that a marked point process^30^ *N* with time-space conditional intensity $\lambda (t,m) = \lambda (t)w(m)$ can faithfully describe the overall activity of observed price changes. $\lambda (t)$ denotes the conditional intensity of the ground process, i.e. of the arrival time process, and *w*(*m*) is the density of the squared returns *m*, also known as the marks of the process. We can interpret this heuristically as $\lambda (t,m)\mathrm {d}t \mathrm {d}m = {\mathbb {E}}[N(\mathrm {d}t \times \mathrm {d}m) \mid {\mathscr {F}}_{t}] = \lambda (t)w(m)\mathrm {d}t \mathrm {d}m$. Then the total conditional intensity of the process above a threshold $\varepsilon$ is found as

10 $$\begin{aligned} \lambda ^ \varepsilon (t) = \int _{ \varepsilon }^{\infty }w(m)\lambda (t)\mathrm {d}m = (1-W( \varepsilon ))\lambda (t), \end{aligned}$$

where $W( \varepsilon ) = {\mathbb {P}}[m \le \varepsilon ]$ is the cumulative distribution corresponding to the density *w*(*m*). We note that the only effect of varying the threshold $\varepsilon$ is to rescale the conditional intensity by the probability that a mark above this threshold is observed. Substituting (10) back into (4), we finally find

11 $$\begin{aligned} \sigma ^2_Y(t) = \lambda (t)\int _{0}^{\infty }(1-W( \varepsilon ))\mathrm {d} \varepsilon = \lambda (t){\bar{m}}, \end{aligned}$$

where ${\bar{m}} = {\mathbb {E}}[m]$. Based on the results from the previous section, which suggested that the predominant clustering mechanism is of endogenous nature across thresholds $\varepsilon$, we take our model for $\lambda (t)$ to be the epidemic model with deterministic exogenous and stochastic endogenous clustering as

12 $$\begin{aligned} \lambda (t) = \mu (t) + \int _0^t\eta (s) h(t-s)\mathrm {d}N(s) = \mu (t) + \sum _{j:t_j < t} \eta (t_j)h(t-t_j). \end{aligned}$$

Note that this is equivalent to usual applications in finance, which assume that the endogenous memory kernel of the marked process can be multiplicatively separated. In this case, the offspring of some point at time $t_j$ with mark $m_j = (Y(t_j) - Y(t_{j-1}))^2$ arrive at Poisson intensity $\phi (t-t_j,m_j) = \rho (m_j)h(t-t_j)$ for some productivity function $\rho (\cdot )$ and we have the correspondence $\eta (t_j) = \rho (m_j)$. The definition of the marks is justified as a consequence of our assumption that $\lambda ^\varepsilon$ can be decomposed as a sum of intensities for different amplitudes of squared returns. Intuitively, a larger return contains more information, or is interpreted by agents as carrying a larger signal, than small returns, so that the future intensity of price changes induced by such a large return depends on its magnitude itself. Moreover, since our estimation scheme extracts the realized triggering owed to the $\eta (t_j)$ directly from the data using the EM algorithm described in the Supplementary Information online, section 1.1, we can indeed be agnostic about the law $m_j \mapsto \eta (t_j)$. Together with (11), the latent variance process is recovered as

13 $$\begin{aligned} \sigma ^2_X(t) = {\bar{m}}\lambda (t) - c(t), \end{aligned}$$

or in terms of integrated quantities

14 $$\begin{aligned} v(t) = {\bar{m}}\Lambda (t) - C(t), \end{aligned}$$

where $\Lambda (t) = \int _0^t\lambda (s)\mathrm {d}s$ is the compensator of *N* and $C(t) = \int _0^tc(s)\mathrm {d}s$.

### Endogenous excess volatility

Using (12) as our model for $\lambda (t)$, and imposing mild conditions on *U*(*t*) as detailed in Supplementary Information online, section 4, the expected integrated variance decomposes as

15 $$\begin{aligned} {\mathbb {E}}\left[ v(t)\right] = ({\bar{m}} - \sigma _e^2)\left[ I(t) + \psi * I(t)\right] , \end{aligned}$$

where $\sigma _e^2$ is the variance of the increments of *U*, $I(t) = \int _{0}^{t} \mu (s) \mathrm {d}s$ and we adopt the notation $a*b(t) = \int _{{\mathbb {R}}} a(t-s)b(s)\mathrm {d}s$ for the convolution of *a* with *b*. The function $\psi$ is often known as the *activity level* in systems with memory^51^ and satisfies the equation $\psi (t) = {\bar{\eta }} h(t) + ({\bar{\eta }} h)*\psi (t)$. The related function $\psi (t) / {\bar{\eta }}$ is often called resolvent, renormalized kernel or response function and describes the response of the system to an impulsive (exogenous) perturbation. See Supplementary Information online, section 3 for technical details and methodological relations to general autoregressive processes. In this epidemic conceptualization of (15), a continuous presence of endogenous excitation $0<{\bar{\eta }}<1$ inflates volatility above its exogenously justified level. The variance of the price process is furthermore additively decomposed into an “efficient” variance, solely driven by exogenous price fluctuations *I*(*t*), and an “excess” volatility. This excess volatility is generated by endogenous feedback effects that follow as responses to exogenous shocks, as made clear by the convolution between $\psi$ and *I* that determines the endogenous excess volatility. We finally note that in fact the full distribution of *v*(*t*) and not only its first moment depends on the distribution of $\lambda (t)$. This distribution was investigated in detail in^52–54^ and exhibits a non-trivial scaling behavior, depending on $\mu$, ${\bar{\eta }}$ and the average time scale of the memory kernel *h*.

As shown in Supplementary Information online, section 4, we can compute a bias-corrected estimator of the integrated variance from (14) as

16 $$\begin{aligned} \begin{aligned} {\hat{v}}(t)&= {\bar{m}}{\hat{\Lambda }}(t) - N(t)\sigma _e^2, \end{aligned} \end{aligned}$$

where we use empirical averages of the observed squared returns to estimate ${\bar{m}}$. An estimate of the exogenous variance follows directly from (15) as

17 $$\begin{aligned} {\hat{v}}^\mu (t) = ({\bar{m}} - \sigma _e^2){\hat{I}}(t). \end{aligned}$$

Finally, we can construct an estimate for the endogenous excess variance by taking the difference between the overall integrated variance (16) and the estimate of the exogenous variance as

18 $$\begin{aligned} {\hat{v}}^\phi (t) = {\hat{v}}(t) - {\hat{v}}^\mu (t). \end{aligned}$$

## Excess volatility in exchange rates and equity futures

We apply the presented methodology to high-frequency data from two major financial markets, the foreign exchange market and the equity futures market. For the former, we consider the exchange rate of the Euro (EUR) against the US-Dollar (USD), which is the most actively traded currency pair globally, and we use one year (2016) of data obtained from its primary interbank trading platform, Electronic Broking Services (EBS). For the equity futures market, we use three months of data from 2019 on the E-mini S&P 500 futures contract, which is one of the most actively traded securities in the world, exclusively on the Chicago Mercantile Exchange (CME). More information on the data sets and their pre-processing can be found in the Supplementary Information online, section 5. The model is calibrated to the data on a daily basis, so that $t \in [0, T]$, for *T* corresponding to one day.

### Results

**Figure 2:**
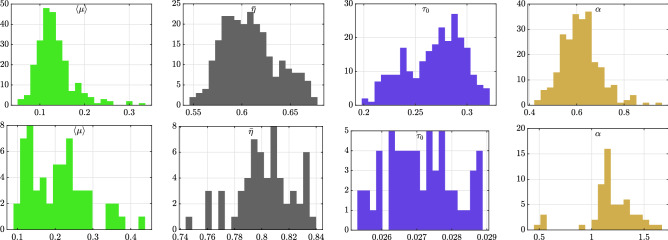
Shows histograms of (daily) parameter estimates of the epidemic model (12). The top row is for the EUR/USD exchange rate and the bottom row for the E-mini futures contract. Shown are, from left to right, the average exogenous intensity $\left<\mu \right>= T^{-1}\int _{0}^{T}\mu (s)\mathrm {d}s$, the branching ratio ${\bar{\eta }}$, the characteristic time scale of the memory kernel $\tau _0$ (in seconds) and its tail index $\alpha$, cf. (8). We note that the bimodal shape of the histogram of $\tau _0$ for the EUR/USD is the signature of a regime-change in the way EBS was disseminating market data to participants^56^, and provides evidence for the ability of the epidemic model to capture low-level characteristics of market activity.

**Figure 3:**
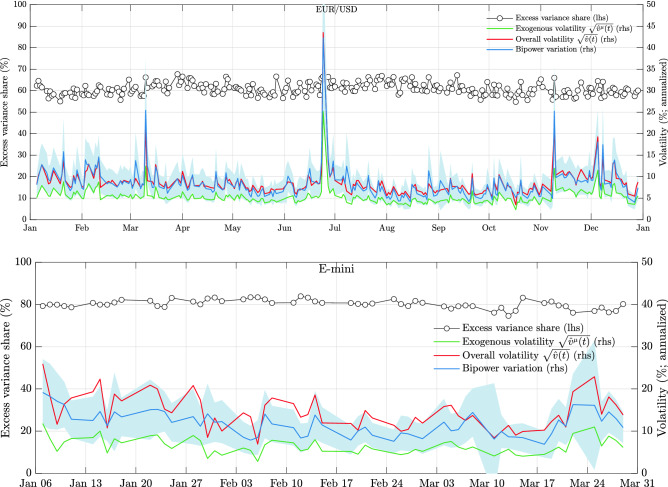
Shows daily estimates of the endogenous excess variance share ($1 - {\hat{v}}^\mu / {\hat{v}}$) on the left axis. Time series of annualized, integrated volatility estimates are plotted on the right axis. The red line indicates the overall volatility estimate according to (16), the green line is an exogenous volatility estimate (17) and the blue line a jump-robust bipower variation estimator^57^ *BV*. The shaded areas are asymptotic 95% confidence intervals for *BV*, indicating good agreement between the estimator (16) and the bipower variation reference. The confidence intervals are computed as $BV \pm z_{0.975}\times \sqrt{{\hat{Q}}/M}$, where ${\hat{Q}}$ is a realized tri-power quarticity estimator^58^ for the integrated quarticity, based on *M* 5-minute returns, and $z_{0.975}$ is the normal inverse cumulative distribution function evaluated at 0.975. All volatility measures have been annualized assuming 252 trading days. The samples are from year 2016 (EUR/USD) and 2019 (E-mini).

As can be seen from the distribution of the parameter estimates of the epidemic model in Fig. 2, the key control parameter of the model ${\bar{\eta }}$ is remarkably stable over time, varying in a range of $\approx 0.1$ around the respective daily means. These means are $\approx 0.6$ for the EUR/USD exchange rate and $\approx 0.8$ for the E-mini futures contract. Based on our calibrations, the system underlying prices is thus highly endogenous, but subcritical, in full agreement with previous studies^33,35,36,55^. According to the branching ratio estimates, endogenous feedback processes play a more important role for equity futures volatility than for foreign exchange at the daily scale. This seems intuitive given that currencies are by their nature more exposed to a constant flow of international news. In support of our methodology to disentangle exogenous from endogenous activity, we also find that spikes in the average exogenous intensity $\left<\mu \right>= I(t) / T$ coincide with days with clear exogenous shocks, such as the US presidential election 2016, or the day of the Brexit referendum. Moreover, as can be seen from Fig. 3, our results suggest that spikes in the volatility in the sample are attributed to spikes in the exogenous volatility component, which then get amplified by a steady presence of feedback mechanisms. For example, on the day following the Brexit referendum (June 24, 2016), the exogenous volatility spikes dramatically from levels around 5% (annualized) to around 25%, while the endogenous excess volatility share remains mostly unchanged. This observation is in excellent agreement with the exogenous nature of the event and a similar observation also applies e.g. to the day of the US elections in November 2016. In order to assess the effectiveness of our bias correction method, Fig. 3 also compares (16) with a bipower variation estimator of the integrated variance^57^. We find that, for the EUR/USD exchange rate, where the estimated observation error is small(er) and exhibits less serial dependence (see Supplementary Information online, section 6. for estimates of the observation error), the bipower variation estimator and ${\hat{v}}(t)$ agree remarkably well on the daily volatility. For the E-mini data, where observation errors are larger and more complex, our estimator appears to yield higher estimates than the bipower variation reference.

Our results thus suggest that endogenous feedback at even the smallest time scales (the characteristic time scales of the endogenous responses are estimated to be of the order of milliseconds) can be responsible for significantly inflated volatility at the macroscopic (daily) scale. In other words, behaviors of agents at very short time scales that are completely unrelated to any change in information about the fundamental value of the asset inflate long-term volatility to the point where it can be far from the fundamental volatility. This effect might even be amplified further by feedback mechanisms that operate at longer time scales. As support for our theory, we remark that the magnitude of excess volatility predicted based on our calibrations is in line with the magnitude that e.g. Shiller deduced in his seminal contribution^14^. Our estimates of the branching ratio for the S&P 500 E-mini futures contract of ${\bar{\eta }}\approx 0.8$ suggest a total volatility that is inflated by a factor of $1/(1-{\bar{\eta }})\approx 5$ compared to the exogenously justified volatility. In^14^, the volatility of the S&P 500 is found to be five to thirteen times too high to be attributed to new information on the fundamental value. Since our approach neglects potential endogenous effects at time scales above one day, it is unsurprising that our estimates of the excess volatility are at the lower end of this range. Moreover, recent reappraisals^59^ of Shiller’s original analysis indicate that S&P 500 excess volatility might be one-third lower when estimated on samples as recent as ours, bringing the estimate even closer to our own results. For exchange rates, our theory predicts magnitudes of the amplification effects that are in excellent agreement with models that explicitly incorporate the dynamics of fundamental values. E.g. in^60^ it is shown that economic fundamentals predict monthly average absolute changes in the EUR/USD exchange rate that are lower by a factor of 2.3 compared to what is observed. Our own estimates with ${\bar{\eta }}\approx 0.6$ suggest that volatility in the EUR/USD exchange rate is inflated by a factor of 2.5. We finally also note that previous estimates for the reflexivity of other financial markets, such as fixed income futures^61^ or cryptocurrencies^61,62^, suggest that our theory and conclusions with respect to the generating mechanism for excess volatility apply very broadly.

### Signatures of market inefficiency

Because our estimation scheme learns a probabilistic version of the branching structure of the epidemic model underlying price fluctuations, we are also able to compute estimates for the quantities $R^1_j$, describing the number of first-generation events triggered independently by some mother event at $t_j$ in the epidemic cascade. The latent fertilities $\eta (t_j)$ from (12) are hereby the means of the distributions of the realized quantities $R^1_j$. Estimating the $R^1_j$ thus allows one to distinguish events that had no direct offspring – either intrinsically due to zero fertility or as a result of randomness – and which events triggered some response. Here we propose that the share of points in some time interval without any apparent offspring can provide a quantitative diagnostic of the degree of efficiency of the market within this interval. This metric decreases with stronger endogenous feedback effects, as measured by the (instantaneous) branching ratio, i.e. the local mean of the fertilities.

Why a larger share of triggering events can be interpreted as an inefficiency of the system deserves some further explanation. A particular property of the epidemic model (12) is that its convergence time after an exogenous shock, i.e. the time it takes for the cascade of responses to die out, is proportional to $(1 - {\bar{\eta }})^{-1}$. As such, the agents in the market take longer and longer to digest some unanticipated news (an exogenous event) as more and more price adjustments produce some reaction in the form of other price fluctuations. The initial exogenous price fluctuation thus gets less and less efficiently processed by the system as the share of triggering events increases. The convergence time of the system finally becomes asymptotically long when the system approaches a state where all events trigger another event (a “critical slowing down”)^63^. At this stage—in other words, when ${\bar{\eta }} \rightarrow 1$—the variance of the event rate also diverges (the “variance of the variance” in our setup). Also the susceptibility of the system, i.e. the response strength to an external perturbation, diverges hyperbolically^64^ and can thus lead to extreme fragility. In this critical regime, the diverging susceptibility of the system is accompanied by the emergence of special properties, such as diverging correlation length, scale-invariant avalanches, power law characterization of the relevant quantities of the system and the absence of a characteristic scale of the response to perturbations. All these properties indicate a growing propensity of the system to break down, which are intimately associated with or revealed by the excess volatility puzzle.

**Figure 4:**
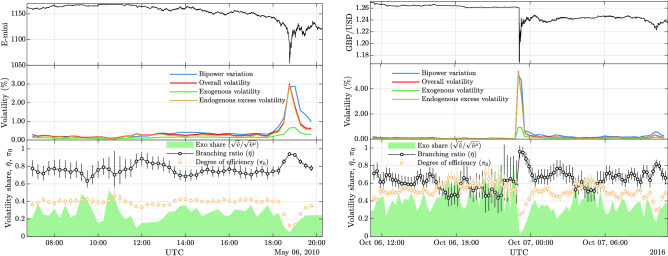
Shows an intraday decomposition of the integrated volatility for two extreme events, computed on overlapping rolling windows of 30 min length and 15 min step size. The left panel shows this decomposition for the E-mini flash crash and the right panel for the crash in the GBP/USD exchange rate. Both events were also analyzed in detail in^61^. Each panel shows in the top row the price evolution in the sample. The middle row shows the endogenous (${\hat{v}}^\phi$ from (18)), exogenous (${\hat{v}}^\mu$ from (17)) and overall (${\hat{v}}$ from (16)) volatility estimates. Also plotted is a bipower variation estimator. The bottom rows plot the exogenous share of volatility according to the model ($\sqrt{{\hat{v}}^\mu } / \sqrt{{\hat{v}}}$). Also plotted is a time-varying estimate of the branching ratio ${\bar{\eta }}$, obtained by averaging the $R^1_j$ within the rolling window, and an estimate for the share of points without offspring $\pi _0 = {\mathbb {P}}[R^1 < 10^{-4}]$, called “degree of efficiency”. The vertical lines indicate 95% confidence intervals for the respective quantities, obtained by bootstrap resampling the $R^1_j$ 1000 times within each interval.

We can illustrate more specifically this interpretation of the share of non-triggering events as a measure of efficiency by looking at instances where markets clearly evolved in a highly irregular and arguably inefficient manner. Specifically, we look at two so-called “flash crashes”—i.e. extreme events where prices exhibit highly irregular and cascading dynamics. The two major events we choose are the flash crash of May 6, 2010 in the S&P 500 E-mini equity futures market and an event in the GBP/USD currency pair on October 7, 2016. As can be seen from Fig. 4, at the trough of the two crashes, 80–90% of all events produced some offspring according to our estimates. The price dynamics are thus to be classified as highly inefficient by this metric. Also the branching ratios at the time of the crashes are found to rise to (near-)criticality. We furthermore remark that the uncertainty in the rolling window estimate of the GBP/USD branching ratio increases drastically, well before the actual crash. Shifts in the variance of state variables due to phase instability are well-known to serve as an early-warning signal of phase transitions in complex systems^65,66^, indicating that indeed the market approached such a transition during the crash. The uncovered inefficiency is mirrored in the time-varying estimates of the endogenous excess volatility. During both events, this excess volatility makes up almost 100% of the overall volatility. The decomposition we perform here thus suggests that there was a clear breakdown in market efficiency at the time of the flash crashes, which led to an excessive divergence in the volatility of price fluctuations as a consequence of destabilizing endogenous feedback effects.

## Discussion

Based on an epidemic model describing the cascade of reactions to price fluctuations, we have constructed a decomposition of financial volatility into an efficient exogenous component and an inefficient excess component. The latter is hereby characterized through self-referential behavior of the agents producing observed price fluctuations. We have shown that, in foreign exchange and equity futures markets, this endogenously induced excess volatility is substantial and appears to be largely stable at longer time scales. Noise amplification by endogenous feedback thus provides an asset- and market-independent explanation for the famous excess volatility puzzle^14^.

It is worth stressing the parsimony of our theory and approach. With only minimal ingredients and without relying on particular and unique economic generating mechanisms usually considered to explain excess volatility—such as competitive interactions between market participants in the presence of noisy information^67^, the possibility of rare disasters^68^, or the irrationality of financial analysts’ expectations^69^—our theory predicts magnitudes of excess volatility which are remarkably close to what the literature has documented in the past. According to our theory, the explanation for the excess volatility puzzle is much simpler than assumed so far: it is a direct consequence of the endogeneity, or reflexivity, of markets and their participants.

We add to the literature in a fundamental manner by providing a microscopic generating mechanism underlying previous studies of excess volatility. For example, typical econometric studies of the phenomenon rely on the ARCH^70^/GARCH^71^ families^72^ of processes. While primarily being thought of as statistical tools, these processes intrinsically describe endogenous, or reflexive, processes. However, they are coarse-grained models of volatility. As a consequence, they do not take into account the discrete and irregularly spaced nature of price fluctuations. We argue that this is a crucial shortcoming as it neglects information on the very mechanism that is responsible for excess volatility: reflexive interactions of (human and machine) agents. These interaction processes are encoded in the times between market events and captured through our point process approach. Using very general forms of GARCH processes, one finds that S&P 500 volatility is a factor of 5 higher than the baseline (exogenous) volatility^73^—in excellent agreement with our estimates. Our results thus give a novel, microscopic interpretation of ARCH-type feedback effects in terms of the noise-amplifying behaviors of financial agents, which lead to excess volatility.

Using our more fine-grained approach, we have also documented that the endogenous excess volatility can vary strongly at shorter, intraday scales, leading to major temporary inefficiencies. Because typical econometric volatility studies using coarse-grained processes by construction impose a minimum time scale that is too large to capture such dynamics, they are also “blind” to these short-term effects. We have argued that these effects are crucial as they are fingerprints of the endogenous nature of excess volatility and should thus be considered with great detail. In particular, we find for example that the bursts in volatility experienced during the flash crashes in the E-mini futures contract on May 6, 2010, and the GBP/USD exchange rate on October 7, 2016, were almost entirely due to endogenous feedback loops inflating this excess volatility component, indicating a significant short-term breakdown of market efficiency.

The finding that noise amplification from endogenous feedback appears to be a generating mechanism for market inefficiencies also bears consequences for system and policy design. In particular, preventive measures and interventions should attempt to identify the relevant feedback mechanisms and then actively break the feedback loops that are causing the undesired inefficiencies. Typical measures aiming to increase resiliency to extreme events by increasing the capacity of institutions/agents to absorb the resulting shocks, e.g. through capital buffers or margins, are ineffective tools in endogenously arising crises.

Several avenues exist to improve upon the presented formalism in future work. First, it is well documented that volatility obeys non-trivial multifractal scaling^27^. The affine approximation in (12) neglects such non-linear phenomena. One way to account more comprehensively for empirical characteristics of volatility is to introduce an exponential non-linearity^54,74,75^ into the conditional intensity (12). Such a fast-accelerating, non-linear intensity can in particular also be expected to provide a better explanation for observed extreme events than the affine forms considered here. Future work in this direction can both draw on and inform other scientific fields, for example the study of stochastic spiking of neurons in computational biology^76^, where such exponential-affine intensities are relevant on both theoretical and empirical grounds^77,78^. A second, even more general avenue of improvement is to discretize the integral of threshold exceedence intensities in (6) and devise separate point process models for each threshold. These intensities can even interact, e.g. cross-excite from large to small return magnitudes, reminiscent of cascade models of financial volatility^79,80^, but expressed in the domain of squared returns.

Finally, while our analysis has focused on financial systems, with the achievement of providing a powerful explanation for the excess volatility puzzle, the *endo-exo* approach followed here can also be very useful in other fields. A key insight of complex systems theory is precisely that apparently different and unrelated systems share underlying universal dynamics. Given that endogenous and exogenous signatures have been observed in many different complex systems, we hypothesize that the principle of feedback at the micro (agent) level propagating to excessively volatile macroscopic (collective) behavior is generically present in systems other than financial markets.

## Supplementary Information

Supplementary Information 1.

## Data Availability

The data from EBS that support the findings of this study can be purchased from the CME Group (https://www.cmegroup.com/trading/market-tech-and-data-services/ebs/data-analytics.html). Tick data for the E-mini futures contract as used in this study can be purchased from Refinitiv Tick History (https://www.refinitiv.com/en/market-data/data-feeds/tick-history) or TickData.com (https://www.tickdata.com/). Restrictions apply to the availability of these data, which were used under license for the current study, and so cannot be shared directly by the authors.
