# Supplementary material for: The excess volatility puzzle explained by financial noise amplification from endogenous feedbacks
Source: Sci Rep. 2022 Nov 7;12:18895. doi: 10.1038/s41598-022-20879-0 (PMC9640597; doi:10.1038/s41598-022-20879-0)
Supplement: Supplementary file 1 — Supplementary Information 1. [file 41598_2022_20879_MOESM1_ESM.pdf]

Supplementary information: The excess volatility puzzle explained by financial noise amplification from endogenous feedbacks

Alexander Wehrli, Didier Sornette

emails: alexander.wehrli@snb.ch, dsornette@ethz.ch

1 Inference scheme for the epidemic model

This section provides an overview of the estimation schemes applied for the different model types used in the main text.

1.1 Description of the estimation algorithm

To estimate the epidemic model, we use an Expectation Maximization (EM) algorithm. Generally, the EM can be used to iteratively build an MLE using the *complete data likelihood function* $L^c(\boldsymbol{\theta}|\boldsymbol{\omega},\mathbf{Z})$[1]. Here, $\boldsymbol{\omega} = \{t_i\}_{i=1,\dots,N(T)}$ denotes the observation times of the events we want to model, in our specific setup these are the times when the observed price changed. $N(T)$ counts the number of events observed on the sample interval of length T. $\boldsymbol{\theta}$ is a vector of model parameters and $\mathbf{Z}$ introduces a set of latent variables which help decompose the estimation into manageable sub-problems. In our specific setup, these latent variables are taken as the branching structure of the epidemic model. Formally, this is encoded as a matrix of indicator variables $\mathbf{Z}_{N(T)\times N(T)} \in \{0,1\}^{N(T)\times N(T)}$, where $Z_{i,j} = 1$ if an event at t_i in the cascade of events is an offspring of an event at $t_j < t_i$. If an event at t_i was exogenously triggered (was an "immigrant" to the system), $Z_{i,i} = 1$. Using the branching structure as the latent information in the EM achieves two important goals. First, it reduces estimation of the epidemic model into separate models for $\mu(t)$ and the offspring processes $R^1 \cdot h(t-s)$[2–4], where R^1 denotes the (random) number of events directly triggered by some other event at a time t after the event at time s. Second, it provides, as a by-product, an estimate for a key object of interest in our study: the branching structure itself.

The EM algorithm presented in the sequel is a modification of the one in[5,6] and extended to deterministically time-varying fertilities in[7]. The modification consists in dropping any assumption regarding a particular model for the fertility in the maximization step of the algorithm and using fully nonparametric offspring estimates per point, based on the branching structure obtained in the expectation step of the EM algorithm. Provided an initial guess of the parameter estimates $\hat{\boldsymbol{\theta}}^{(0)}$, the EM algorithm iterates the expectation step (E-step) and maximization step (M-step) in each iteration l until $\hat{\boldsymbol{\theta}}^{(l)}$ converges[8]. In the E-step, the algorithm computes the expected value of $\ell^c(\boldsymbol{\theta}) = \log L^c(\boldsymbol{\theta}|\boldsymbol{\omega},\mathbf{Z})$ with respect to the conditional distribution of the latent variables $\mathbf{Z}$, given the observed data and the current parameter estimates

$$Q(\boldsymbol{\theta}|\boldsymbol{\omega},\hat{\boldsymbol{\theta}}^{(l)}) = \mathbb{E}_{\mathbf{Z}|\boldsymbol{\omega},\hat{\boldsymbol{\theta}}^{(l)}}[\ell^c(\boldsymbol{\theta})]. \tag{1}$$

The subsequent M-step maximizes (1) to obtain new parameter estimates

$$\hat{\boldsymbol{\theta}}^{(l+1)} = \arg\max_{\boldsymbol{\theta}} Q(\boldsymbol{\theta}|\boldsymbol{\omega},\hat{\boldsymbol{\theta}}^{(l)}). \tag{2}$$

For the specific model of the main text, with conditional intensity $\lambda(t) = \mu(t) + \sum_{j:t_j<t}\eta(t_j)h(t-t_j)$, the estimation proceeds as follows:

1. *Initialization*: Initialize the parameter vector $\hat{\boldsymbol{\theta}}^{(0)} = \{\mu(t),(R^1_j)_{i=1}^{N(T)},h(t)\}$ with some sensible starting values. Set $l = 1$ and start the EM.

2. *E-step*: In the expectation step, produce an estimate of the underlying branching structure $\pi_{i,j} = \mathbb{P}[Z_{i,j} = 1 \mid \boldsymbol{\omega},\hat{\boldsymbol{\theta}}^{(l-1)}]$. At the l-th iteration of the algorithm, this can be obtained using random thinning[9] as

$$\pi_{i,i} = \frac{\mu(t_i|\hat{\boldsymbol{\theta}}^{(l-1)})}{\lambda(t_i|\hat{\boldsymbol{\theta}}^{(l-1)})} \quad \text{and} \quad \pi_{i,j} = \frac{R^1_j h(t_i - t_j|\hat{\boldsymbol{\theta}}^{(l-1)})}{\lambda(t_i|\hat{\boldsymbol{\theta}}^{(l-1)})} \quad \text{for} \quad i > j. \tag{3}$$

3. *M-step*: In the maximization step, new values of the parameters $\hat{\boldsymbol{\theta}}^{(l)}$ are obtained by maximizing the expected complete data log-likelihood, $\ell^c(\boldsymbol{\theta})$, defined as

$$\ell^c(\boldsymbol{\theta}) = \underbrace{\sum_{i=1}^{N(T)} \pi_{i,i} \log \mu(t_i) - \int_0^T \mu(t)\mathrm{d}t}_{\ell^\mu} + \underbrace{\sum_{i=1}^{N(T)} \sum_{j:t_j<t_i} \pi_{i,j} \log(R_j^1 h(t_i - t_j)) - \sum_{j=1}^{N(T)} R_j^1 H(T - t_j)}_{\ell^h}, \tag{4}$$

where $H(t) = \int_0^t h(s)\mathrm{d}s$ is the cumulative distribution corresponding to the offspring density h. Assuming that μ and h do not share any parameters, the two parts ℓ^μ and ℓ^h can be maximized separately. Furthermore, we obtain estimates for the R_j^1's from the branching structure as

$$\hat{R_j^1} = \frac{\sum_{i>j} \pi_{i,j}}{H(T - t_j|\hat{\boldsymbol{\theta}}^{(l)})}. \tag{5}$$

4. *Check convergence*: Stop estimation if $||\hat{\boldsymbol{\theta}}^{(l)} - \hat{\boldsymbol{\theta}}^{(l-1)}||_\infty$ is below some small threshold, or go back to the E-step for another iteration $l = l + 1$ otherwise.

An estimate of the branching ratio of the epidemic process can finally obtained as

$$\bar{\eta} = \frac{1}{N(T)} \sum_{j=1}^{N(T)} \hat{R}_j^1. \tag{6}$$

In our empirical calibrations, we stop the EM algorithm if the convergence criterion is satisfied at a threshold of 10^{-4} for a few consecutive iterations, or if a maximum number of 500 iterations was performed.

1.2 Adaptive estimation of $\mu(t)$

To facilitate a flexible and systematic estimation of the exogenous driving process μ, we use flexible logspline[10] estimators to control for trends and shocks in the data in an adaptive fashion[5]. The flexibility and smoothness provided by these modern, non-parametric estimators has been found to be crucial in controlling for the significant exogenous heterogeneity in high-frequency financial data[6]. To select models, we vary the degrees of freedom allowed for $\mu(t)$ in $p \in \{0, 3, 6, 10, 15, 20, 25, 30, 40, 50, 60\}$, where $p = 0$ denotes a model with constant exogenous driving $\mu(t) \equiv \mu > 0$, compute log-likelihoods

$$\ell(\hat{\boldsymbol{\theta}}^{(l)}) = \sum_{i=1}^{N(T)} \log \lambda(t_i|\hat{\boldsymbol{\theta}}^{(l)}) - \int_0^T \lambda(s|\hat{\boldsymbol{\theta}}^{(l)})\mathrm{d}s \tag{7}$$

and select the best model for $\mu(t)$ according to the Bayesian Information Criterion (BIC) $-2\ell(\hat{\boldsymbol{\theta}}^{(l)}) + \log(N(T))p$.

1.3 Simulation study of the EM estimator

We test the ability of the proposed EM procedure to characterize the distribution of the R_j^1 in a simulation study. For this purpose, we simulate realizations of Hawkes processes with constant immigration $\mu = 0.1$, a branching ratio of $\bar{\eta} = 0.6$ and three different fertility distributions $f(\eta)$, namely:

1. Exponentially distributed fertilities $f_\lambda(\eta) = \lambda e^{-\lambda x}$ with $\lambda = 1/\bar{\eta}$.
2. Weibull distributed fertilities $f_{\lambda,\beta}(\eta) = \beta\lambda(\lambda\eta)^{\beta-1}e^{-(\lambda\eta)^\beta}$ with $\lambda = \bar{\eta}/2$ and $\beta = 0.5$.
3. Power law distributed fertilities $f_\gamma(\eta) = \gamma\eta_0^\gamma(\eta + \eta_0)^{-(1+\gamma)}$ with $\eta_0 = 0.3$ and $\gamma = 1.5$.

We use a simple exponential offspring density with ground truth $h(t) = 2e^{-2t}$ in all three cases. Fitting is then done using the EM on 100 realizations of length $T = 86400$ seconds from each of these three processes. The results from this simulation study are summarized in Figure S1. First, we notice that the branching ratios $\bar{\eta}$ recovered by the EM are quite consistent, with estimates ≈ 0.62 for all three fertility distributions considered. Regarding the exact shape of the distributions, we find that the head of the distribution of R^1 appears difficult to estimate – not surprising given that the true distribution is discrete, whereas the estimator is real-valued. Regarding the tail, we see that even though there is clearly some bias, the shape of the tail is approximated quite well. In particular, the tail of the distribution recovered by the EM exhibits approximate power

law dynamics in the case where the underlying true fertility distribution also follows a power law – the heaviness of the tail is however underestimated. In the case of exponential fertility, the EM estimates also clearly drop off exponentially in the tail. Therefore, we conclude that, while inferring the exact distribution of the fertilities directly from the EM estimates of the number of first generation offspring is difficult, testing for certain properties of the underlying distribution (e.g. computing its mean or conditioning it on some observable, testing for power law tail, asserting the share of zero-offspring points) works well.

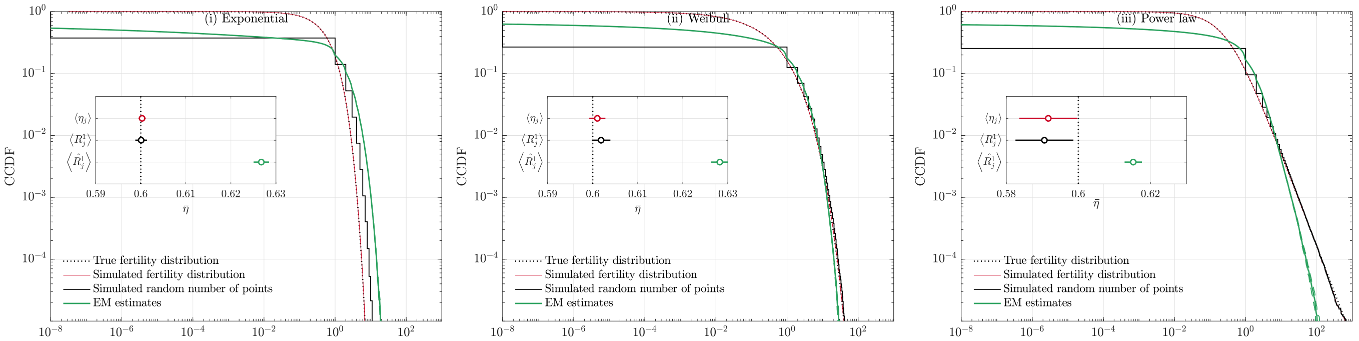

Figure S1. Shows the empirical complementary cumulative distribution (CCDF) of the simulated fertilities (η_j), the corresponding true random number of first generation offspring (R_j^1) and the EM estimates thereof ($\hat{R}_j^1$), for the three simulation cases considered. The dashed green lines indicate 95% lower and upper confidence bounds for the CCDF of the EM estimates, obtained from Greenwood's formula. In the inset, the average of the three quantities across the 100 process realizations of length $T = 86400$ seconds, are compared to the true value (the mean $\bar{\eta}$ of the respective fertility distribution; dashed vertical line). The horizontal whiskers indicate 95% confidence intervals for the mean obtained by bootstrap resampling 100 times.

1.4 Extension of the estimation algorithm in the presence of exogenous stochastic clustering

Here, we give a short practical description of the extended algorithm required to estimate the model in the presence of stochastic exogenous clustering for the special case where $\gamma(s) \equiv \bar{\gamma}$ and $\eta(s) \equiv \bar{\eta}$, i.e. with conditional intensity

$$\lambda(t) = \mu(t) + \int_0^t \bar{\gamma} g(t-s) \mathrm{d}N^{\mu}(s) + \int_0^t \bar{\eta} h(t-s) \mathrm{d}N(s). \tag{8}$$

The algorithm synthesizes the principles described above for the generalized Hawkes model, with the MCEM algorithm as derived in[11] for the inclusion of the additional shot-noise effects.

For the estimation of a model like (8), or (7) of the main text, the missing data has to be extended to $(\mathbf{Z}, C)$, where C denotes the immigrant process induced by N^{μ}, and the complete data log-likelihood thus takes the form

$$Q(\boldsymbol{\theta}|\boldsymbol{\omega}, \hat{\boldsymbol{\theta}}^{(l)}) = \mathbb{E}_{\mathbf{Z},\mathrm{C}|\boldsymbol{\omega},\hat{\boldsymbol{\theta}}^{(l)}}[\log L^c(\boldsymbol{\theta}|\boldsymbol{\omega}, \mathbf{Z}, C)], \tag{9}$$

which can be approximated by averaging over a sample $\boldsymbol{c}^{(k)}$, $k = 1, ..., K$ of immigrant realizations from a density $\propto p(\boldsymbol{c}|\boldsymbol{\omega}, \hat{\boldsymbol{\theta}}^{(l)})$ as

$$Q(\boldsymbol{\theta}|\boldsymbol{\omega}, \hat{\boldsymbol{\theta}}^{(l)}) \approx \frac{1}{K} \sum_{k=1}^{K} \mathbb{E}_{\mathbf{Z}|\boldsymbol{\omega},\hat{\boldsymbol{\theta}}^{(l)},\boldsymbol{c}^{(k)}}[\log L^c(\boldsymbol{\theta}|\boldsymbol{\omega}, \mathbf{Z}, \boldsymbol{c}^{(k)})]. \tag{10}$$

To obtain a conditional MCMC sample from the immigrant process, a Metropolis-Hastings algorithm is employed. Given some state $\boldsymbol{c}^{(k)}$ of the Markov chain, each Metropolis-Hastings iteration starts with randomly proposing the birth or death of an immigrant. In case we choose birth, we uniformly select some $c^* \in \boldsymbol{\omega} \setminus \boldsymbol{c}^{(k)}$. The Metropolis-Hastings birth ratio to decide whether c^* is accepted, given some immigrant vector $\boldsymbol{c}$, is obtained from

$$\begin{aligned} r_b(\boldsymbol{c}, c^*) = & \frac{N(T) - |\boldsymbol{c}|}{|\boldsymbol{c}| + 1} \mu(c^*) e^{-\bar{\gamma} G(T - c^*)} \times \\ & \prod_{t_i \in \boldsymbol{\omega} \setminus (\boldsymbol{c} \cup c^*)} \left(1 + \frac{\bar{\gamma} g(t_i - c^*)}{\lambda(t_i|\boldsymbol{c}) - \mu(t_i)}\right) / \left(\lambda(c^*|\boldsymbol{c}) - \mu(c^*)\right). \end{aligned} \tag{11}$$

and we accept $\boldsymbol{c}^{(k+1)} = \boldsymbol{c}^{(k)} \cup c^*$ with acceptance probability $\min\{r_b(\boldsymbol{c}^{(k)}, c^*), 1\}$. In case we chose death, $c^* \in \boldsymbol{c}^{(k)}$ is removed from the current state of the Markov chain with acceptance probability $\min\{1/r_b(\boldsymbol{c}^{(k)} \setminus c^*, c^*), 1\}$. The Metropolis-Hastings iterations can then be repeated until a sufficiently long chain is available, and the desired K samples be drawn from this chain.

Then, given some $\boldsymbol{c}^{(k)} = (c_1^k, ..., c_{n_\mu^k}^k)$ and due to the fact that the process maps to a branching process, the inner expectation in (10) decouples analogously to (4), using branching probabilities which can be evaluated due to random thinning[9] in a similar fashion like (3) as

$$\pi_{i,j}^{g,k} = \begin{cases} \frac{\bar{\gamma} g(t_i - c_j^k | \hat{\boldsymbol{\theta}}^{(l)})}{\lambda(t_i | \hat{\boldsymbol{\theta}}^{(l)}, \boldsymbol{c}^{(k)}) - \mu(t_i | \hat{\boldsymbol{\theta}}^{(l)})} & \text{for } t_i \in \boldsymbol{\omega} \setminus \boldsymbol{c}^{(k)}, t_j \in \boldsymbol{\omega} \\ 0 & \text{otherwise} \end{cases}$$
$$\pi_{i,j}^{h,k} = \begin{cases} \frac{\bar{\eta} h(t_i - t_j | \hat{\boldsymbol{\theta}}^{(l)})}{\lambda(t_i | \hat{\boldsymbol{\theta}}^{(l)}, \boldsymbol{c}^{(k)}) - \mu(t_i | \hat{\boldsymbol{\theta}}^{(l)})} & \text{for } t_i \in \boldsymbol{\omega} \setminus \boldsymbol{c}^{(k)}, t_j \in \boldsymbol{\omega} \\ 0 & \text{otherwise.} \end{cases} \tag{12}$$

In the M-step of the algorithm, the objective (10) is maximized, which – as for estimation without shot-noise effects – has the structure of decoupled density estimation. First, $\mu(t)$ is estimated on the MCMC sample of immigration times $\{\boldsymbol{c}^{(1)}, ..., \boldsymbol{c}^{(K)}\}$. Next, the offspring density $h(\cdot)$ is estimated with objective ℓ^h from (4), with the difference that the offspring probabilities used in the objective function need to be averaged over the MCMC samples as

$$\pi_{i,j}^h = \frac{1}{K} \sum_{k=1}^K \pi_{i,j}^{h,k}. \tag{13}$$

Estimation of $g(\cdot)$ finally requires to pool inter-event times and weights over all MCMC samples $\boldsymbol{\pi}^g := \{(\pi_{i,j}^{g,k}, t_i - c_j^k)\}_{k=1,...,K}$ and maximize

$$\ell_g^c(\boldsymbol{\theta} | \boldsymbol{\pi}^g) = \frac{1}{K} \sum_{k=1}^K \sum_{i=1}^{N(T)} \sum_{j=1}^{n_\mu^k} \pi_{i,j}^{g,k} \log\left(\bar{\gamma} g(t_i - c_j^k)\right) - \frac{\bar{\gamma}}{K} \sum_{k=1}^K \sum_{j=1}^{n_\mu^k} G(T - c_j^k), \tag{14}$$

where $G(t) = \int_0^t g(s) \mathrm{d}s$ is the cumulative distribution corresponding to the exogenous offspring density $g(t)$. Estimating nested models can be done by enforcing the restrictions $\bar{\gamma} = 0$ and/or $\bar{\eta} = 0$[12]. For the applications presented in the main text, the MCEM estimation is done with $K = 50$ samples taken uniformly from the second half of a chain of length 100000, generated by the Metropolis-Hastings algorithm presented here.

1.5 Model selection in the presence of exogenous stochastic clustering

In the absence of stochastic exogenous clustering ($\gamma \equiv 0$), model selection is possible consistently using information criteria (see section 1.2), because the unconditional likelihood is accessible. If however $\gamma > 0$, then conditional likelihoods must be computed using MCMC as discussed in[13]. To fix notation, denote by $df(\cdot)$ the degrees of freedom of a function, thus we have the exogenous flexibility as $p = df(\mu)$ and the endogenous flexibility of the model as $r = df(h) + df(g)$. Then $\hat{\boldsymbol{\theta}}_{p,r}$ denotes the parameter estimates of a particular model with overall $p + r$ degrees of freedom.

To compute conditional likelihoods, we create an MCMC sample $\boldsymbol{c}^{(1)}, \boldsymbol{c}^{(2)}, ..., \boldsymbol{c}^{(k)}, ..., \boldsymbol{c}^{(K)}$ of immigrant realizations with density $\propto p(\boldsymbol{c} | \boldsymbol{\omega}, \hat{\boldsymbol{\theta}}_{p,r})$ – just like in the E-step of the MCEM algorithm in the previous section – being the density of an immigrant realization $\boldsymbol{c}$, conditional on the parameter estimates $\hat{\boldsymbol{\theta}}_{p,r}$ and the data $\boldsymbol{\omega}$. Each $\boldsymbol{c}^{(k)}$ defines a conditional log-likelihood

$$\ell(\hat{\boldsymbol{\theta}}_{p,r} | \boldsymbol{\omega}, \boldsymbol{c}^{(k)}) = \sum_{i=1}^{N(T)} \log \lambda(t_i | \boldsymbol{c}^{(k)}, \hat{\boldsymbol{\theta}}_{p,r}) - \int_0^T \mu(s | \hat{\boldsymbol{\theta}}_{p,r}) \mathrm{d}s - \sum_{i=1}^{N(T)} \eta H(T - t_i | \hat{\boldsymbol{\theta}}_{p,r}) - \sum_{i=1}^{n_\mu^k} \gamma G(T - c_i^k | \hat{\boldsymbol{\theta}}_{p,r}) \tag{15}$$

and a conditional BIC

$$\mathrm{BIC}(\hat{\boldsymbol{\theta}}_{p,r} | \boldsymbol{\omega}, \boldsymbol{c}^{(k)}) = -2\ell(\hat{\boldsymbol{\theta}}_{p,r} | \boldsymbol{\omega}, \boldsymbol{c}^{(k)}) + \log(N(T))(p + r). \tag{16}$$

In order to select models, we apply a two-step procedure:

1. For each model type – represented with a given r – we first select the optimal degrees of freedom p_r^* for the immigration intensity μ. For this purpose, for $b = 1, ..., B$ and $p = 0, ..., P$, we sample $j_b^p \in [1, K]$ to obtain a bootstrap sample

$$\mathscr{B}_{p,b} = \left\{\mathrm{BIC}(\hat{\boldsymbol{\theta}}_{p,r} | \boldsymbol{\omega}, \boldsymbol{c}^{(j_b^p)})\right\}_{p \in \{0,...,P\}, b \in \{1,...,B\}}. \tag{17}$$

We can then compute conditionally (on the particularly sampled immigrant realizations) optimal degrees of freedom $p_r^b = \arg\min_p \mathscr{B}_{p,b}$, being the optimal degrees of freedom per bootstrap sample, according to the randomly chosen conditional BICs. The final choice across bootstrap samples is then $p_r^* = \text{mode}(\{p_r^b\}_{b=1,\dots,B})$.

2. To compare two different models, say r' and r'', we sample pairs (i_b, j_b) from $[1,K] \times [1,K]$, again $b = 1,\dots,B$ times, and then select r' over r'' on a given bootstrap sample b if

$$\text{BIC}(\hat{\boldsymbol{\theta}}_{p^*_{r'},r'}|\boldsymbol{\omega},\boldsymbol{c}^{(i_b)}) < \text{BIC}(\hat{\boldsymbol{\theta}}_{p^*_{r''},r''}|\boldsymbol{\omega},\boldsymbol{c}^{(j_b)}). \tag{18}$$

We can then finally compute selection probabilities $P(r',r'') = B^{-1}\sum_{b=1}^{B} \mathbb{1}_{\{\text{BIC}(\hat{\boldsymbol{\theta}}_{p^*_{r'},r'}|\boldsymbol{\omega},\boldsymbol{c}^{(i_b)})<\text{BIC}(\hat{\boldsymbol{\theta}}_{p^*_{r''},r''}|\boldsymbol{\omega},\boldsymbol{c}^{(j_b)})\}}$, giving an estimate of the likelihood that a certain model type r' is to be preferred over another model type r''.

For all applications in the main text, we choose $B = 1000$ and perform the test on an MCMC sample of $K = 100$ draws from a Markov chain of length $300'000$.

2 Endogenous vs. exogenous clustering

In order to test which of the clustering mechanisms are dominant in practice, we conduct the following test. For some randomly selected days of data we calibrate models with constant branching coefficients $\gamma(s) \equiv \bar{\gamma}$, $\eta(s) \equiv \bar{\eta}$ and the adaptive estimator using logsplines from section 1.2 for $\mu^{\varepsilon}(t)$, which also allows for deterministic clustering through time-variation in $\mu^{\varepsilon}(t)$. We then estimate increasingly complex models, starting from no stochastic clustering (Deterministic Exo: $\bar{\gamma} = 0$, $\bar{\eta} = 0$), to include only exogenous clustering (Stochastic Exo: $\bar{\eta} = 0$), or deterministic exogenous and stochastic endogenous clustering (Stochastic Endo: $\bar{\gamma} = 0$), or finally a mixture of all three clustering mechanisms (Stochastic Endo-Exo). Additionally, we allow h to either exhibit short- (exponential) or heavy-tailed (approximate power law[14]) memory.

In Table S1, we illustrate how frequently (across samples and thresholds ε) a certain clustering mechanism is selected according to the procedure described in section 1.5. We observe first that the most frequently selected model is a model

	EUR/USD				E-mini			
	Deterministic Exo	Stochastic Exo (ST)	Stochastic Endo (ST)	Stochastic Endo-Exo (ST)	Deterministic Exo	Stochastic Exo (ST)	Stochastic Endo (ST)	Stochastic Endo-Exo (ST)
Stochastic Exo (ST)	0%				17%			
Stochastic Endo (ST)	100%	100%			97%	80%		
Stochastic Endo-Exo (ST)	60%	100%	0%		97%	80%	17%	
Stochastic Endo (HT)	100%	100%	100%	100%	97%	80%	65%	77%

Table S1. Shows a summary of the model selection procedure described in section 1.5, applied to three randomly chosen days of data (EUR/USD: Aug 9, 2016; Sep 30, 2016, Oct 3, 2016; E-mini: Feb 8, 2019, Feb 26, 2019, Mar 7, 2019). The ε are chosen as the (5%, 10%, ..., 95%)-quantiles of the distribution of squared returns. The offspring densities $g(t)$ and $h(t)$ are modelled using an exponential distribution for short-tailed (ST) models, and using an approximate Pareto distribution[14] for heavy-tailed (HT) models. The branching coefficients are taken as constants. The table shows the selection frequency of the respective row-model over the corresponding column-model, pooled across all thresholds ε and samples.

with deterministic exogenous clustering and stochastic endogenous clustering (mechanisms (A) and (C) from the main text). Moreover, an endogenous offspring distribution with heavy tail is preferred over short-tailed memory. A more detailed summary of the estimates for the stochastic endo model with heavy-tailed memory, being the most frequently selected model, is additionally given in Figure S2.

3 First-order properties and relation to autoregressive time series

A string of recent literature has developed[15–17] and reviewed[18] first- and second-order statistical properties of self-excited / Hawkes point processes in the context of financial applications, which were originally formulated in[19,20] and their behavior

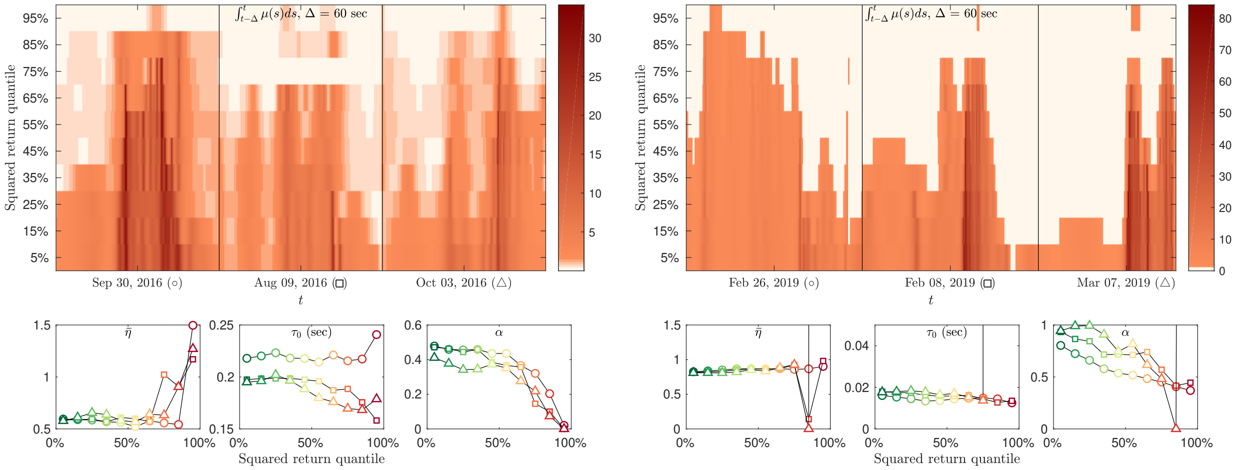

Figure S2. Shows a summary of the exemplary estimates for the stochastic endo model ($\gamma(s) \equiv 0$) with heavy-tailed (approximate Pareto[14] with tail index α) memory from Table S1, for the EUR/USD (left panel) and the E-mini (right panel) data. The heatmap in the top of each panel shows the integrated exogenous intensity $\int_{t-\Delta}^{t} \mu(s)\mathrm{d}s$, with Δ equal to one minute (cf. also the unconditionally expected activity profiles in Figure 1 of the main text), across various thresholds ε corresponding to the (5%, 10%, ..., 95%)-quantiles of the distribution of squared returns. The smaller plots below the heatmaps show, also as a function of ε, estimates of the branching ratio $\bar{\eta}$, the characteristic time scale τ_0 and the tail index α of the memory kernel $h(t)$.

characterized carefully and in great detail even before the recent spur of interest from the quantitative finance literature[21–26]. Here we build on these results to derive the time-dependent properties of first-order characteristics of the epidemic process underlying the study in the main text. In a few extensive comments, we furthermore highlight some important parallels to the analysis of autoregressive time series.

In the following, we consider the conditional intensity $\lambda(t) = \mu(t) + \sum_{i:t_i<t} \bar{\eta} h(t-t_i) = \mu(t) + \int_0^t \bar{\eta} h(t-s)\mathrm{d}N(s)$ after having averaged over the distribution of fertilities. With some abuse of notation, we denote this averaged intensity also as $\lambda(t)$. Taking the expectation of $\lambda(t)$, we find the expected intensity as the solution to the equation

$$\mathbb{E}[\lambda(t)] = \mu(t) + \int_0^t \phi(t-s)\,\mathbb{E}[\lambda(s)]\mathrm{d}s, \tag{19}$$

for $\phi(t) = \bar{\eta} h(t)$ and $\bar{\eta} = \mathbb{E}[\eta]$. Equation (19) has the form of a classical renewal equation and can be expressed as the convolution

$$e(t) = \mu(t) + \phi * e(t) \tag{20}$$

for $e(t) = \mathbb{E}[\lambda(t)]$. Denoting the Laplace transform of a function $x(t)$ as $\widehat{x}(z) = \int_0^\infty e^{-zt} x(t)\mathrm{d}t$, the solution of (20) is given in the Laplace domain as

$$\widehat{e}(z) = \frac{\widehat{\mu}(z)}{1 - \widehat{\phi}(z)}. \tag{21}$$

If we define the auto-convolution of ϕ for $n \geq 1$ as $\phi^{*(n+1)}(t) = \int_0^t \phi(t-s)\phi^{*n}(s)\mathrm{d}s = \phi * \phi^{*n}(t)$ with $\phi^{*1} = \phi$ and $\phi^{*0} = 1$, we note that

$$\phi^{*n}(t) = \bar{\eta}^n h^{*n}(t). \tag{22}$$

Since h is a probability density function, h^{*n} itself is the cumulative distribution of a sum of n independent random variables, each with density h. By explicitly calculating the L^1-norm of h^{*n} and remarking that $\int_0^\infty \mathrm{d}t \int_0^t \mathrm{d}s = \int_0^\infty \mathrm{d}s \int_s^\infty \mathrm{d}t$, it can be shown that the L^1-norm of $h^{*n}(t)$ is equal to the L^1-norm of $h^{*(n-1)}(t)$. By recurrence, it is also equal to the norm of $h(t)$ and consequently $\phi^{*n}(t)$ has L^1-norm equals $\bar{\eta}^n$ for all n. Thus the auxiliary function ψ^0, defined as

$$\psi^0(t) = \sum_{n=0}^{\infty} \phi^{*n}(t) \tag{23}$$

is also in L^1, given $\bar{\eta} < 1$, as

$$\int_0^\infty \psi^0(t)\mathrm{d}t = \sum_{n=0}^{\infty} \bar{\eta}^n = \frac{1}{1-\bar{\eta}} < \infty. \tag{24}$$

To finally find the solution of (20), we see that $\psi^0(t)$ has Laplace transform

$$\widehat{\psi^0}(z) = \sum_{n=0}^{\infty} (\widehat{\phi}(z))^n = \frac{1}{1-\widehat{\phi}(z)}. \tag{25}$$

Note that the infinite sum in (25) resembles closely the characteristic polynomial of an MA(∞) process. To see this, define the lag operator L as $L^s y_t = y_{t-s}$ and consider the AR(p) process $y_t = -\sum_{j=1}^{p} \phi_j L^j y_t + \varepsilon_t$ with innovations $\varepsilon_t \sim IID(0, \sigma_\varepsilon^2)$. Then the characteristic polynomial $\Phi(\eta) = \sum_{n=0}^{p} \phi_j \eta^n$ – defined on $|\eta| < 1$ – expresses the innovations as $\varepsilon_t = \Phi(L)y_t$ and assuming we can invert the polynomial, we get the equivalent moving-average form $y_t = \Phi^{-1}(L)\varepsilon_t = \Psi(L)\varepsilon_t$, where $\Phi(L)\Psi(L) = 1$. In the specific case of an AR(1) process, the characteristic polynomial is $\Phi(L) = 1 - \eta L$ and thus the characteristic polynomial of the moving-average representation in the time series case has an obvious conceptual correspondence in the point process case as

$$\Psi(L) = \sum_{n=0}^{\infty} \eta^n L^n \quad \Longleftrightarrow \quad \widehat{\psi^0}(z) = \sum_{n=0}^{\infty} \eta^n (\widehat{h}(z))^n. \tag{26}$$

In the Hawkes process case, we have a similar equivalence regarding the "characteristic polynomials" as $(1-\widehat{\phi}(z))(1+\widehat{\psi}(z)) = 1$. Furthermore, the indicated comparison to the Laplace transform of ψ^0 can be interpreted such that – as intuitively reasonable – the "lag operator" in this moving average representation of the Hawkes process is given by the Laplace transform of the offspring density $\widehat{h}(z)$, which precisely defines the probability of lagged event occurrences given some mother event. Furthermore, the integrability condition in (24) is equivalent to the condition in MA(∞) models where the absolute MA-coefficients must be summable for y_t to take finite values and for the process not to explode. The similarities to time series analysis are conceptually striking: in order to learn about the probability structure of the Hawkes process (the autoregressive point process), we have ended up looking for an equivalent moving-average representation. But when working with data, we prefer the simple structure of the Hawkes. In time series analysis, when dealing with data, autoregression as a linear method is often very convenient and computationally fast. But when evaluating the structural properties of a process, they can often more easily be obtained via the moving-average representation – one example being the autocovariance function, which can be obtained from the convolution of the moving-average coefficients. Continuing the derivation, the expression for $\widehat{\psi^0}(z)$ from (25) can be inserted into (21) to find the solution of the renewal equation in the time domain by applying the inverse Laplace transform $\mathscr{L}^{-1}$ as

$$\begin{aligned} e(t) &= \mathscr{L}^{-1}\left[\widehat{\mu}(z)\sum_{n=0}^{\infty}(\widehat{\phi}(z))^n\right] = \mathscr{L}^{-1}\left[\sum_{n=0}^{\infty}(\hat{\phi}(z))^n\right] * \mu(t) \\ &= \left(\sum_{n=0}^{\infty}\mathscr{L}^{-1}\left[(\widehat{\phi}(z))^n\right]\right) * \mu(t) = \left(\sum_{n=0}^{\infty}\phi^{*n}(t)\right) * \mu(t) \\ &= \left(1+\sum_{n=1}^{\infty}\phi^{*n}(t)\right) * \mu(t) \\ &= \mu(t) + \psi * \mu(t) \end{aligned} \tag{27}$$

where $\psi(t) = \sum_{n\geq 1}\phi^{*n}(t)$, which is in turn defined by its Laplace transform

$$\widehat{\psi}(z) = \frac{\widehat{\phi}(z)}{1-\widehat{\phi}(z)}. \tag{28}$$

We note that since $\phi(t)$ is positive and supported by $\mathbb{R}^+$, and $\psi(t)$ is simply defined as the sum of convolutions of $\phi(t)$, $\psi(t)$ is also positive and supported by $\mathbb{R}^+$.

The mapping of the Hawkes process to a Galton-Watson branching process[27] gives valuable intuition on the derivations at this point. We recall that the number of direct offspring of any point in the genealogy of the mapped branching process has Poisson distribution with mean $\bar{\eta}$. The expected size of the n-th generation of the equivalent branching process is thus $\bar{\eta}^n$ (cf.

(22)). Further, the total number of individuals in the entire cluster formed by the branching process, including the immigrant (0-th generation), has a Borel distribution, having mean $(1-\bar{\eta})^{-1}$ (cf. (25)) and variance $\bar{\eta}(1-\bar{\eta})^{-3}$. The expected size of the total offspring over all generations, triggered by one immigrant is $\bar{\eta}/(1-\bar{\eta})$, cf. (28). As such, we see that $\widehat{\psi^0}(0)$ and $\widehat{\psi}(0)$ are the expected value of the random variables representing respectively the cluster size including and without the triggering immigrant[1].

Using this fact, the well-known result for the average intensity in the case of constant immigration $\mu(t) \equiv \mu$ follows in a straight-forward manner as

$$\mathbb{E}[\lambda(t)] = \mu + \mu * \psi(t) = \mu + \mu \int_{\mathbb{R}^+} \psi(s)\mathrm{d}s. \tag{29}$$

By virtue of (28), this translates into

$$\mu + \mu\widehat{\psi}(0) = \mu + \mu \frac{\widehat{\phi}(0)}{1-\widehat{\phi}(0)} = \frac{\mu}{1-\bar{\eta}}. \tag{30}$$

In the time domain, the function $\psi(t)$ satisfies the equation

$$\psi(t) = \phi(t) + \phi * \psi(t) \tag{31}$$

and is often known as the “activity level” in systems with memory – in the language of branching processes the dispersion of all offspring of one immigrant over time. The function $R(t) = \psi(t)/\bar{\eta} = h * \psi^0(t)$ is often called resolvent, renormalized kernel or response function and describes the response of the system to an impulsive (exogenous) perturbation. It integrates to $1/(1-\bar{\eta})$, representing the mean cluster size of the Galton-Watson branching process to which the Hawkes process can be mapped. Note again the link to time series analysis: the impulse-response function (to a unit shock in the innovations) in an AR(p) process is given by the corresponding MA(∞) coefficients. Analogously – as already noted earlier – the function $\psi^0(t)$ (or $\psi(t)$ when not counting the immigrant) defines the activity of the Hawkes process following an immigrant, i.e. the response to an innovation.

In the case of an exponential offspring density, $\psi(t)$ is known explicitly

$$\phi(t) = \bar{\eta}\beta e^{-\beta t} \quad \Rightarrow \quad \psi(t) = \bar{\eta}\beta e^{-\beta(1-\bar{\eta})t}, \tag{32}$$

which illustrates the name “renormalized kernel” as the characteristic time scale of the kernel is renormalized with $(1-\bar{\eta})$, namely the characteristic time scale is amplified by the factor $1/(1-\bar{\eta})$, thus expressing the increased duration of the cluster due to the cascade of generations.

Of particular interest in many applications are kernels with power law memory

$$\phi(t) = \bar{\eta}\frac{\alpha\tau_0^\alpha}{(\tau_0+t)^{1+\alpha}} \quad \Rightarrow \quad \widehat{\phi}(z) = \bar{\eta}\alpha e^{\tau_0 z}(\tau_0 z)^\alpha \Gamma(-\alpha, \tau_0 z) \tag{33}$$

for $\Gamma(a,x)$ the upper incomplete gamma function. In this case, the Laplace transform in (28) cannot be inverted analytically, but the asymptotic properties of the resulting resolvent have been studied in detail as well, see[25].

To compute the expected number of points up to some time t, we can use the solution (27) to obtain

$$\mathbb{E}[N(t)] = \int_0^t \mathbb{E}[\lambda(s)]\mathrm{d}s = \int_0^t \mu(s)\mathrm{d}s + \int_0^t (\psi * \mu(s))\mathrm{d}s. \tag{34}$$

For the case of constant exogenous intensity, the fact that $\psi * \mu = \mu\widehat{\psi}(0)$ directly recovers $\mathbb{E}[N(t)] = \mu t/(1-\bar{\eta})$.

For the case of time-dependent immigration, we notice that, by the Fubini theorem, the integral of the endogenous part of the compensator $\Lambda(t) = \int_0^t \lambda(s)\mathrm{d}s$ satisfies

$$\int_0^t \mathrm{d}s \int_0^s \phi(s-u)\mathrm{d}N(u) = \int_0^t \mathrm{d}N(u)\int_u^t \phi(s-u)\mathrm{d}s = \bar{\eta}\int_0^t H(t-s)\mathrm{d}N(s) \tag{35}$$

[1] For example, we can compute the average duration of a cluster as $\int_0^\infty t \frac{\psi^0(t)}{\int_0^\infty \psi^0(s)\mathrm{d}s}\mathrm{d}t$, which e.g. in the case of an exponential kernel like in (32) yields $\frac{1}{\beta(1-\bar{\eta})}$.

and integration by parts[2] with $H(t)$ shows that this last expression equals $\phi * N(t)$ and thus we get $\Lambda(t) = I(t) + \phi * N(t)$ for $I(t) = \int_0^t \mu(s)\mathrm{d}s$. Taking expectations, a renewal equation for the expected event count is found, which is similar to (20), as

$$\mathbb{E}[N(t)] = I(t) + \phi * \mathbb{E}[N(t)], \tag{36}$$

where we have used the fact that, at fixed t, $N(t)|\Lambda(t) \sim \mathrm{Poi}(\Lambda(t))$ and thus $\mathbb{E}[N(t)] = \mathbb{E}[\Lambda(t)]$. The solution of this equation is analogously

$$\mathbb{E}[N(t)] = I(t) + \psi * I(t). \tag{37}$$

As a final remark underlining the conceptual similarities between autoregressive point processes and time series, it is worth noting that the scaling limits of the Hawkes process derived in[28,29] also find their natural analogue in the time series case[30].

4 Effect of observation error on variance estimates of X and bias correction

A general result from stochastic calculus says that for a random partition[3] of $[0,t]$, $\pi = \{0 = t_0 \leq \ldots \leq t_n \leq t\}$, and two general semimartingales W and Z, we have that[31]

$$\operatorname{plim} \sum_{i:t_i \leq t} \left(W(t_i) - W(t_{i-1})\right)\left(Z(t_i) - Z(t_{i-1})\right) = [W,Z]_t \tag{38}$$

and the convergence in probability to the quadratic covariation $[\cdot,\cdot]_t$ is for the mesh size $\sup_{i \geq 1}(t_i - t_{i-1}) \to 0$ as $n \to \infty$. Furthermore, in the case of the specific dynamics we postulate for our fundamental price X, the fundamental identity for stochastic integrals[4] yields

$$[X,X]_t = \int_0^t \sigma_X^2(s)\mathrm{d}[B,B]_s = \int_0^t \sigma_X^2(s)\mathrm{d}s = v(t). \tag{39}$$

As a consequence, a common way to estimate the integrated variance of a process is to use the sum of squared returns, or *realized variance*. Assuming we are able to observe the latent process X without error at times t_i, the ideal estimator

$$\sum_{i:t_i \leq t} \left(X(t_i) - X(t_{i-1})\right)^2 \tag{40}$$

converges to $v(t)$ in probability as the sampling frequency increases[32]. However, in the presence of observation error, we are forced to consider instead

$$[Y,Y]_t = [X,X]_t + 2\,[X,U]_t + [U,U]_t\,, \tag{41}$$

so the sum of squared observed returns is in general inconsistent for $v(t)$.

This argument immediately makes clear that σ_Y^2 from equation (4) in the main text, based on observed returns, is subject to the same biases as the realized variance estimator (40), as soon as there is observation error. We make the following assumptions:

1. The noise process U is stochastically independent of X. This is a standard assumption in the literature, even though there is some evidence for more intricate noise dynamics at tick frequencies[33].
2. $U(t)$ is a covariance stationary process with $\mathbb{E}[U(t)] = 0$.
3. We sample the processes at the times t_i where the observed price changes to compute our variance estimates.

Under assumptions 1. - 3. and defining $e_i = U(t_i) - U(t_{i-1})$ with $\mathbb{E}[e_i^2] = \sigma_e^2$, the bias in equation (14) from the main text is found by taking expectations as

$$\mathbb{E}[\bar{m}\Lambda(t) - v(t)] = \mathbb{E}[[U,U]_t] = \sum_{i=1}^{N(t)} \mathbb{E}[e_i^2] = N(t)\sigma_e^2. \tag{42}$$

Furthermore, under the assumptions made, (42) makes clear that the *relative* contributions of the exogenous and endogenous variances from $\Lambda(t)$ are unchanged by the observation error – only their *absolute* magnitudes are inflated when using observed returns, due to the presence of U.

---

[2] We can write $H(0)N(t) - H(T)N(0) = \int_0^t H(t-s)\mathrm{d}N(s) - \int_0^t h(t-s)N(s)\mathrm{d}s$

[3] It should be noted that, so as to not complicate notation, we have omitted that in fact we are dealing with an increasing sequence of partitions, so that $n \to \infty$.

[4] For a semimartingale process X and an X-integrable process ξ, we have the identity $[\int \xi \mathrm{d}X, \int \xi \mathrm{d}X] = \int \xi^2 \mathrm{d}[X]$.

5 Data sets and pre-processing

The main data sets used here are mid-prices of two highly liquid markets. First, we study the EUR/USD currency pair traded on Electronic Broking Services (EBS), one of the major interbank markets for foreign exchange and key market place for the EUR/USD currency pair. The time stamps of the EBS data set have a resolution of 100ms, and there is an entry whenever either the best bid, offer or both changed within the last 100ms interval. We use data for the entire calendar year of 2016. During this period, there were around 30'000 mid-price changes per day on average. Since foreign exchange markets operate continuously, we partition each week of the data into five 24 hour trading days, starting at the market opening on Sunday evening and disregard the 48h weekend period as there is no activity during that time.

The second main data set we use is from the S&P 500 E-mini futures contract traded on the Globex electronic platform at the Chicago Mercantile Exchange (CME), considered a key venue for equity price discovery in the United States. Trading on the Globex platform is possible almost continuously – only halted temporarily between 3:15pm and 3:30pm CT (Chicago Time) and for a maintenance window between 4pm and 5pm CT. We work with three months of data from January to March 2019 and define each trading day to start at 5pm CT and end at 3:15pm CT, discarding the 30 minutes of trading between the end of the temporary trading halt and the start of the maintenance window. This results in around 85'000 mid price changes per trading day on average in the sample. The time stamps of the E-mini mid-price changes have millisecond resolution.

For some intraday case studies, we will additionally revisit a data set for the GBP/USD currency pair from[7], traded on Thomson Reuters Matching (now known as Refinitiv FX), which was obtained from Refinitiv Tick History and also has millisecond resolution.

As customary for point process calibration[14,34], we randomize the time stamps of all data sets within an interval of uncertainty (here 100 milliseconds) and exclude any holidays from the analysis.

6 Estimation of the observation error

To complete our variance estimator requires specification of a particular model for the observation error. Under the common assumption of a serially independent noise process U with variance $\mathbb{E}[U^2] = \omega_1^2$, the increments e_i follow an MA(1) structure. In this case, an estimate for the observation error variance can be obtained from the relation $\mathbb{E}[y_i y_{i-1}] = -\omega_1^2$[35,36] as

$$\hat{\omega}_1^2 = -\frac{1}{N(t)-1}\sum_{i=2}^{N(t)} y_i y_{i-1}, \tag{43}$$

where $y_i = Y(t_i) - Y(t_{i-1})$. Under the independent observation error assumption, the bias correction term thus becomes $\sigma_e^2 = 2\hat{\omega}_1^2$. To account for more complex serial dependence in the observation error, we will consider the generalization of e_i to an MA(q) structure as

$$e_i = \sum_{j=1}^{q} \rho_j (\nu_{i+1-j} - \nu_{i-j}), \tag{44}$$

where $\nu_i \sim IID(0, \omega_q^2)$ and $\rho_1 = 1$ for identification purposes. In this case, the bias correction term is

$$\sigma_e^2 = 2\omega_q^2 \left(\sum_{j=1}^{q} \rho_j^2 - \sum_{j=2}^{q} \rho_j \rho_{j-1} \right). \tag{45}$$

Estimation of the noise model (45) can be done using the quasi maximum likelihood (QML) approach of[37]. This procedure allows one to select the order q of the MA model consistently using information criteria. Here, we rely on the Akaike Information Criterion (AIC), which was shown to yield consistent estimates in[37]. Important for our purposes, the QML scheme also provides estimates for the autocovariance function of the noise process, which can then be factorized to estimate the ρ_j.

Estimates for $\bar{m}$ and the observation error variance according to the different specifications in the previous subsection are presented in Table S2. The fact, that $\hat{\omega}_1^2$ is frequently found to be negative, indicates significant departure from the standard i.i.d. assumption for the observation error. This is especially pronounced for the E-mini price process, but also happens for EUR/USD prices. This pattern is also confirmed by the AIC-selected orders of the MA(q) observation error model, where the median selected order is 23 for the E-mini and 8 for the EUR/USD. Finally, when accounting for dependence in the observation error, its variance ω_q^2 is approximately one order of magnitude larger for the E-mini than for the EUR/USD, suggesting a more significant role of observation error in E-mini variance estimation. For empirical applications presented in the main text, the bias correction applied is (45).

	$\bar{m}$	$\hat{\omega}_1^2$	q^{AIC}	$\hat{\omega}_q^2$
E-mini	0.20617	-0.00033	23	0.01923
	(0.19473, 0.23518)	(-0.01202, 0.03213)	(6, 50)	(0.00038, 0.04569)
EUR/USD	0.09306	0.00427	8	0.00156
	(0.07671 0.14265)	(-0.00672, 0.01578)	(3, 50)	(0.00001,0.01136)

Table S2. Shows the median daily estimates of the respective quantities defined in previous subsections and (5%, 95%)-quantiles in brackets below. The units of the return parameters are in bp^2. q^{AIC} indicates the AIC-optimal order of the MA(q) noise (45) according to the QML procedure in[37].

References

1. McLachlan, G. & Krishnan, T. *The EM algorithm and extensions*. Wiley series in probability and statistics (John Wiley & Sons, 2008), 2nd edn.
2. Marsan, D. & Lengline, O. Extending Earthquakes' Reach Through Cascading. *Science* **319**, 1076–1079, DOI: 10.1126/science.1148783 (2008).
3. Veen, A. & Schoenberg, F. Estimation of space-time branching process models in seismology using an EM-type algorithm. *J. Amer. Stat. Assoc., 103* 614–624, DOI: 10.1198/016214508000000148 (2008).
4. Lewis, E. & Mohler, G. A nonparametric EM algorithm for multiscale Hawkes processes. *J. Nonparametric Stat.* (2011).
5. Wheatley, S., Wehrli, A. & Sornette, D. The endo-exo problem in high frequency financial price fluctuations and rejecting criticality. *Quant. Finance* **19**, 1165–1178, DOI: 10.1080/14697688.2018.1550266 (2020).
6. Wehrli, A., Wheatley, S. & Sornette, D. Scale-, time- and asset-dependence of Hawkes process estimates on high frequency price changes. *Quant. Finance* **21**, 729–752, DOI: 10.1080/14697688.2020.1838602 (2021).
7. Wehrli, A. & Sornette, D. Classification of flash crashes using the Hawkes(p,q) framework. *Quant. Finance* **22**, DOI: 10.1080/14697688.2021.1941212 (2021).
8. Wu, C. On the convergence properties of the EM algorithm. *The Annals Stat. 11.1* 95–103, DOI: 10.1214/aos/1176346060 (1983).
9. Ogata, Y. On Lewis' simulation method for point processes. *IEEE Transactions on Inf. Theory* **27**, 23–31, DOI: 10.1109/TIT.1981.1056305 (1981).
10. Kooperberg, C. & Stone, C. J. A study of logspline density estimation. *Comput. Stat. & Data Analysis* **12**, 327–347, DOI: 10.1016/0167-9473(91)90115-I (1991).
11. Schatz, M., Wheatley, S. & Sornette, D. The ARMA Point Process and its Estimation. *Econom. Stat. in press* DOI: 10.1016/j.ecosta.2021.11.002 (2021).
12. Kim, D. K. & Taylor, J. M. The restricted EM algorithm for maximum likelihood estimation under linear restrictions on the parameters. *J. Am. Stat. Assoc.* **90**, 708–716, DOI: 10.1080/01621459.1995.10476564 (1995).
13. Møller, J. & Waagepetersen, R. *Statistical Inference and Simulation for Spatial Point Processes*. Monographs on Statistics and Applied Probability 100 (Chapman & Hall/CRC: Boca Raton, FL, 2003).
14. Hardiman, S. J., Bercot, N. & Bouchaud, J. P. Critical reflexivity in financial markets: A Hawkes process analysis. *Eur. Phys. J. B* **86**, 442, DOI: 10.1140/epjb/e2013-40107-3 (2013).
15. Bacry, E., Dayri, K. & Muzy, J.F. Non-parametric kernel estimation for symmetric Hawkes processes. Application to high frequency financial data. *Eur. Phys. J. B* **85**, 157, DOI: 10.1140/EPJB/E2012-21005-8 (2012).
16. Bacry, E., Delattre, S., Hoffmann, M. & Muzy, J. Some limit theorems for Hawkes processes and application to financial statistics. *Stoch. Process. Appl.* **123**, 2475–2499, DOI: 10.1016/j.spa.2013.04.007 (2013).
17. Bacry, E. & Muzy, J.-F. First- and Second-Order Statistics Characterization of Hawkes Processes and Non-Parametric Estimation. *IEEE Transactions on Inf. Theory* **62**, 2184–2202, DOI: 10.1109/TIT.2016.2533397 (2016).
18. Bacry, E., Mastromatteo, I. & Muzy, J.-F. Hawkes processes in finance. *Mark. Microstruct. Liq.* **1**, 1550005, DOI: 10.1142/S2382626615500057 (2015).

19. Hawkes, A. Spectra of some self-exciting and mutually exciting point processes. *Biometrika* **58**, 83–90, DOI: 10.1093/biomet/58.1.83 (1971).

20. Hawkes, A. Point Spectra of Some Mutually Exciting Point Processes. *J. Royal Stat. Soc. Ser. B (Methodological)* **33**, 438–443, DOI: 10.1111/j.2517-6161.1971.tb01530.x (1971).

21. Sornette, D. & Sornette, A. Renormalization of earthquake aftershocks. *Geophys. Res. Lett.* **26**, 1981–1984, DOI: 10.1029/1999GL900394 (1999).

22. Helmstetter, A. & Sornette, D. Subcritical and supercritical regimes in epidemic models of earthquake aftershocks. *J. Geophys. Res. Solid Earth* **107**, ESE 10–1 – ESE 10–21, DOI: 10.1029/2001JB001580 (2002).

23. Helmstetter, A. & Sornette, D. Diffusion of epicenters of earthquake aftershocks, Omori's law, and generalized continuous-time random walk models. *Phys. Rev. E* **66**, 061104, DOI: 10.1103/PhysRevE.66.061104 (2002).

24. Sornette, D. & Helmstetter, A. Endogeneous Versus Exogeneous Shocks in Systems with Memory. *Phys. A* **318**, 577–591, DOI: 10.1016/S0378-4371(02)01371-7 (2003).

25. Saichev, A. & Sornette, D. Generation-by-generation dissection of the response function in long memory epidemic processes. *The Eur. Phys. J. B-Condensed Matter Complex Syst.* **75**, 343–355, DOI: 10.1140/epjb/e2010-00121-7 (2010).

26. Saichev, A., Maillart, T. & Sornette, D. Hierarchy of Temporal Responses of Multivariate Self-Excited Epidemic Processes. *Eur. Phys. J. B* **86**, 1–19, DOI: 10.1140/epjb/e2013-30493-9 (2013).

27. Hawkes, A. & Oakes, D. A Cluster Process Representation of a Self-Exciting Process. *J. Appl. Probab.* **11**, 493–503, DOI: 10.2307/3212693 (1974).

28. Jaisson, T. & Rosenbaum, M. Limit theorems for nearly unstable Hawkes processes. *The Annals Appl. Probab.* **25**, 600–631, DOI: 10.1214/14-AAP1005 (2015).

29. Jaisson, T. & Rosenbaum, M. Rough fractional diffusions as scaling limits of nearly unstable heavy tailed Hawkes processes. *The Annals Appl. Probab.* **26**, 2860–2882, DOI: 10.1214/15-AAP1164 (2016).

30. Jaisson, T. & Rosenbaum, M. The Different Asymptotic Regimes of Nearly Unstable Autoregressive Processes. In Podolskij, M., Stelzer, R., Thorbjørnsen, S. & Veraart, A. E. D. (eds.) *The Fascination of Probability, Statistics and their Applications: In Honour of Ole E. Barndorff-Nielsen*, 283–301, DOI: 10.1007/978-3-319-25826-3_13 (Springer International Publishing, Cham, 2016).

31. Protter, P. E. *Stochastic Integration and Differential Equations* (Springer-Verlag Berlin Heidelberg, 2005), 2nd edn.

32. Barndorff-Nielsen, O. & Shephard, N. Estimating quadratic variation using realized variance. *J. Appl. Econom.* **17**, 457–477, DOI: 10.1002/jae.691 (2002).

33. Hansen, P. R. & Lunde, A. Realized Variance and Market Microstructure Noise. *J. Bus. & Econ. Stat.* **24**, 127–161, DOI: 10.1198/073500106000000071 (2006).

34. Filimonov, V. & Sornette, D. Quantifying reflexivity in financial markets: Toward a prediction of flash crashes. *Physical Review E* **85**, 056108, DOI: 10.1103/PhysRevE.85.056108 (2012).

35. Oomen, R. C. Comment on 2005 JBES invited address "Realized variance and market microstructure noise" by Peter R. Hansen and Asger Lunde. *J. Bus. Econ. Stat.* **2**, 195–202 (2006).

36. Oomen, R. C. A. Properties of Realized Variance under Alternative Sampling Schemes. *J. Bus. & Econ. Stat.* **24**, 219–237, DOI: 10.1198/073500106000000044 (2006).

37. Da, R. & Xiu, D. When Moving-Average Models Meet High-Frequency Data: Uniform Inference on Volatility. *Econometrica* **89**, 2787–2825, DOI: 10.3982/ECTA15593 (2021).
